# Assessing Acrylic Inks: A Study of Colorants and Lightfastness

**DOI:** 10.1021/acsomega.6c04091

**Published:** 2026-09-22

**Authors:** Teodora Raicu, Matea Krmpotić, Iva Bogdanović Radović, Zdravko Siketić, Katja Sterflinger, Dubravka Jembrih-Simbürger

**Affiliations:** † Institute of Natural Sciences and Technology in the Arts (INTK), 27261 Academy of Fine Arts Vienna, Kelsenstrasse 2, Vienna A-1030, Austria; ‡ Division of Experimental Physics, Laboratory for Ion Beam Interactions, Ruđer Bošković Institute, Bijenička cesta 54, Zagreb HR-10000, Croatia

## Abstract

Acrylic inks, derived from acrylic emulsion paints, have become increasingly favored by contemporary artists. These inks have advanced traditional watercolor techniques by improving water resistance and color intensity. This work presents the first systematic study on acrylic inks, focusing on synthetic organic pigments (SOPs) and their photodegradation behavior. A minimally invasive analytical protocol, requiring no chemical sample treatment, was developed on acrylic ink-on-paper mock-ups for direct SOP identification, combining μ-Raman spectroscopy and secondary ion mass spectrometry with MeV primary ions (MeV SIMS). Pyrolysis–gas chromatography/mass spectrometry (Py-GC/MS) was applied to selected mock-ups for binder and additive identification, which also influenced photodegradation. The examined acrylic inks were originally used by the Austrian caricaturist Erich Sokol (1933–2003) and manufactured by Schmincke, Rotring, and Winsor & Newton. Mock-ups were aged using a microfading tester (MFT) under LED illumination and analyzed before and after aging. For comparison, selected unaged ink mock-ups were displayed for one year under actual museum conditions. Despite the manufacturers’ claims of lightfastness, both procedures (MFT aging and museum display) revealed chemical changes in several SOPs. Results show SOP mixtures present in most acrylic inks. In some instances, one pigment within a mixture appeared to degrade faster than the others—e.g., PY13 relative to PY83 in yellow inks, or PY14 relative to PG36 and PY74 in green inks. The applied approach was verified on a minute sample from one of Sokol’s caricatures, demonstrating its effectiveness for minimally invasive SOP identification in modern and contemporary works on paper.

## Graphical abstract

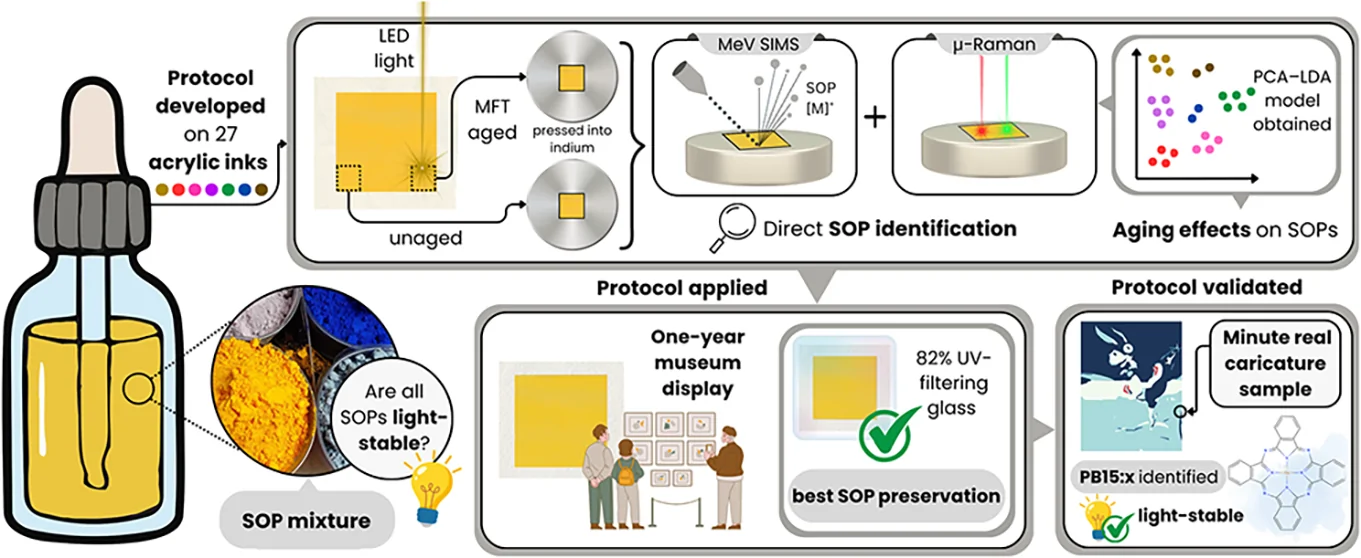

## 1 Introduction

It is difficult to imagine modern art without works like Mark Rothko’s *“No. 61 (Rust and Blue)”* (1953) and Andy Warhol’s *“Campbell’s Soup Cans”* (1961–62), whose intense colors and distinctive textures were made possible by the introduction of acrylic emulsion paints in the early 1950s.^1^ Valued for its ease of handling, elasticity, fast drying, non-toxicity, and wide availability, acrylic emulsion paint quickly became a favorite among artists.^2,3^ While it can be thinned with water to enhance fluidity, excessive dilution weakens its binding capacity, limiting its suitability for watercolor and airbrushing techniques. To meet the demand for a pigment-rich yet fluid medium, manufacturers introduced acrylic inks in the 1960s.^4^ These are ultra-fluid acrylic emulsion paints that combine the permanence of traditional acrylic paints with the workability of inks. Acrylic inks outperform conventional watercolors, as they dry to form a water-resistant film.

Despite their popularity, no patents or technical reports describing acrylic ink formulations for artistic use are accessible to the general public. However, these materials likely share a similar composition to acrylic emulsion paints, consisting primarily of acrylate and/or methacrylate homo- or copolymers stabilized by surfactants, among other additives.^5^ Manufacturers often note full compatibility between acrylic inks and acrylic emulsion paints,^6,7^ supporting this assumption. According to Golden,^8^ acrylic inks contain only acrylic polymer dispersions as binders, with viscosity controlled by the ratio of monomers in the polymer chain, rather than water dilution. Glass transition temperature (*T*_g_) governs polymer flexibility, which must be low enough to ensure film flexibility upon application but high enough to prevent cracking upon drying.^9,10^ Common monomers in acrylic emulsions are *n*-butyl acrylate (nBA), ethyl acrylate (EA), and methyl acrylate (MA), resulting in soft polymers.^10^ In contrast, the methyl methacrylate (MMA) monomer forms a hard polymer and is often copolymerized with nBA, EA, and/or MA monomers in controlled ratios to fine-tune the mechanical properties. It can be hypothesized that acrylic inks contain similar copolymers but with a lower ratio of MMA content to ensure the formation of lower-*T*_g_ copolymers, thereby maintaining the soft, fluid consistency required of inks.

Pigment loadings in acrylic inks are similar to those in acrylic emulsion paints, with particle size being the main distinction. Compared to paints, inks typically contain pigments that are finely milled to sub-micron particle sizes to achieve low viscosities.^11,12^ Patents for acrylic emulsion paints and acrylic-based writing inks^13–15^ report pigment concentrations (%w/w) of ∼5–10%, with an upper limit of 20% for highly fluid systems, as higher pigment loadings significantly reduce flow.^15^ Color charts from major manufacturers (e.g., Liquitex, Golden) reveal a preference for synthetic organic pigments (SOPs), notably phthalocyanines, diarylides, and quinacridones, which are chosen for their brilliance, fine particle size, and good dispersion and stability.^16^ Inorganic pigments such as titanium white and carbon black are used less frequently, as their opacity counteracts the watercolor-like effects often sought by artists.

While acrylic emulsion paints have been extensively studied, the composition and photoaging behavior of acrylic inks remain underexplored. However, analytical methodologies developed for acrylic emulsion paints can be adapted for inks. Acrylic emulsion paints have been investigated using various spectroscopic and mass spectrometry techniques. Among the most widely used is Fourier transform infrared (FTIR) spectroscopy in attenuated total reflectance (ATR) mode, enabling SOP and binder identification, but only when present in well-defined ratios in two-component acrylic paint mock-ups.^17–19^ However, inks are easily absorbed into the paper substrate, and since FTIR-ATR analyzes a few micrometers in depth,^20^ the resulting spectra can be dominated by cellulose bands, obscuring signals from other chemical compounds. Raman spectroscopy complements FTIR by effectively identifying SOPs regardless of substrate.^19,21,22^ This is because most SOPs have chromophores (e.g., −N=N– bond found in azo pigments) that present strong Raman scattering.^23,24^ In SOP pigment mixtures, Raman often detects only the most Raman-active colorant(s), with other SOPs present evading detection.^25^ Pyrolysis–gas chromatography mass spectrometry (Py-GC/MS) has proven effective for characterizing acrylic copolymers and key additives such as surfactants, defoamers, humectants, and rheology modifiers in acrylic emulsion paints.^5,26–28^ However, it is destructive, as the sample is pyrolyzed during analysis.^28^ Moreover, SOP identification is challenging as markers appear only at specific pyrolyzing temperatures (e.g., green phthalocyanine pigments need >800 °C).^29^ Since binders, additives, and SOPs require different optimal pyrolyzing temperatures, multiple samples are needed for analysis, which is not feasible for limited and valuable samples from artworks. To address this, direct thermally resolved mass spectrometry (DTMS) and laser desorption mass spectrometry (LDI-MS) have been employed, offering clear identification of both SOPs and binders present in acrylic emulsion paints.^30^ However, DTMS is also destructive and typically allows identification of a single pigment in mixtures.^31^

Due to the strict limitations imposed by curators on sampling artworks on paper such as caricatures painted with acrylic inks, it is crucial to employ non-destructive or minimally invasive analytical methods to prevent any noticeable material loss. For SOP analysis, the combination of μ-Raman spectroscopy with secondary ion mass spectrometry with MeV primary ions (MeV SIMS) is chosen as both techniques can be applied on minute samples without chemical treatment or sample consumption.^32^ While confocal μ-Raman primarily gives information from the first 1–2 μm of the sample, MeV SIMS probes the uppermost surface layer (<10 nm) by desorbing secondary ions. Owing to the dominance of the electronic stopping regime, MeV SIMS enables relatively soft ionization of organic molecules, producing molecular ions or (de)­protonated SOP molecules, as well as larger fragment ions, even in complex mixtures, as demonstrated in our previous studies.^25,33–35^ The combined μ-Raman together with subsequent statistical evaluation of the spectra and MeV SIMS is thus expected to provide a detailed understanding of pigment composition and photoaging in acrylic inks. For artificial photoaging, microfading testing (MFT) is chosen, a minimally invasive technique developed in the 1990s for cultural heritage applications, which can be applied directly to the art objects/mock-ups.^36,37^ MFT focuses a small high-intensity light spot (≤0.5 mm in diameter; Xenon or LED light) onto the object/sample surface, while a spectrophotometer measures the reflected light to track color changes in real time. Photosensitivity is thus determined by calculating the color difference (Δ*E*) from the initial state, with results compared to the ISO Blue Wool (BW) scale. In use since 1914, the BW scale has been widely employed to examine the fading proneness of colorant-containing objects like drawings, documents, paintings, and textiles. The scale comprises eight reference strips of wool dyed with colorants of known light sensitivity, ranging from the highly sensitive BW1 (containing Acid Blue 109) to the very stable BW8 (containing Vat Blue 8).^37,38^ A light dose of ∼0.3 Mlux·h induces a perceptible chromatic change in BW1, whereas BW8 requires ∼1100 Mlux·h.^37^

In this study, an analytical protocol using μ-Raman and MeV SIMS for identifying SOPs in acrylic ink mock-ups was developed with the original inks of Erich Sokol (1933–2003), a famous Austrian caricaturist, and applied to a sample from one of his caricatures. To evaluate SOP light stability, acrylic ink mock-ups were photoaged using microfading testing (MFT) with LED light. Unaged mock-ups were selected for display in a temporary museum exhibition (*“I LOVE DEIX”*, 17 February 2024–02 February 2025, Karikaturmuseum Krems, Austria) for comparison purposes. For understanding aging impact, subsequent statistical evaluation of Raman data using principal component analysis (PCA) for data reduction and linear discriminant analysis (LDA) for classification and discrimination was performed. Binders and additives influencing photodegradation were characterized with Py-GC/MS on selected mock-ups.

Given the widespread use of acrylic inks and their presence in museum collections, understanding the long-term light stability of their constituent SOPs is of great importance. Despite the manufacturers’ claim of lightfastness, thin ink layers applied to paper may be highly vulnerable to photodegradation, an issue this study also aimed to clarify for conservators and curators.

## 2 Materials and Methods

### 2.1 Mock-Ups

This study included two sets of acrylic ink mock-ups: one on Japanese paper (Subsection 2.1.1.) and another on watercolor paper (Subsection 2.1.2.). Ink mock-ups on Japanese paper (90 g/m^2^, Nebel KG, Austria) were used to develop the analytical protocol for identifying SOPs in acrylic inks and examine their stability after MFT aging. Japanese paper was selected as the substrate because it contains no fillers that could interfere with the analysis. Ink mock-ups on watercolor paper (300 g/m^2^, lignin-free, FABRIANO, Italy) were designed to mimic actual caricatures and displayed alongside original caricatures during a one-year museum exhibition. Additionally, the watercolor paper substrate allowed for an assessment of the analytical approach on filler-containing paper substrates.

#### 2.1.1 Acrylic Ink Mock-Ups on Japanese Paper

Twenty-seven acrylic inks from original ink bottles used by Erich Sokol (1933–2003) were selected based on colors most frequently observed in his caricatures: yellow, red, magenta, violet, green, blue, and brown. All ink bottles belong to the artist’s estate kept at the Landessammlungen NÖ (State Collections of Lower Austria). The inks were manufactured by Schmincke (AEROCOLOR), Rotring (ArtiColor and A.C.P.), and Winsor & Newton (W&N). Inks with identical color names were sometimes found in bottles with different labels, indicating production at different times and potential formulation changes. These inks were assigned a common code followed by a number (e.g., the three bottles labeled *“Zinnoberrot” (cinnabar red)* were designated S324(1), S324(2), and S324(3)). For applying inks onto Japanese paper, eyedroppers built into the ink bottle caps were used, facilitating easy application. An overview of ink bottles and mock-ups is provided in Table 1. Images of original acrylic ink bottles (with labels) and their corresponding mock-ups can be found in Supporting Information (SI), Figures S1 and S2.

**Table 1:** List of Analyzed Mock-Ups with Acrylic Inks on Japanese Paper, Including Ink Color, Assigned Ink Code, Commercial Name on the Bottle Label, and Company and Trademark

**general assigned color**	**ink assigned code**	**commercial ink code**	**commercial name (original bottle label in German and English translation)**	**company & trademark**
**yellow**	S230	28 320	Indischgelb (indian yellow)	Schmincke AEROCOLOR
	RACGY		Goldgelb (golden yellow)	Rotring ArtiColor
	RACY		Gelb (zitron) (lemon yellow)	Rotring ArtiColor
**red**	S324(1)	28 324	Zinnoberrot (cinnabar red)	Schmincke AEROCOLOR
	S324(2)	28 324	Zinnoberrot (cinnabar red)	Schmincke AEROCOLOR
	S324(3)	28 324	Zinnoberrot (cinnabar red)	Schmincke AEROCOLOR
	S330	28 330	Scharlachrot (scarlet red)	Schmincke AEROCOLOR
	RACR		Rot (red)	Rotring ArtiColor
**magenta**	S340(1)	28 340	Karmin (carmine)	Schmincke AEROCOLOR
	S340(2)	28 340	Karminrot (carmine red)	Schmincke AEROCOLOR
	S344	28 344	Krapprosa (rose madder)	Schmincke AEROCOLOR
	S350(1)	28 350	Magentarot (magenta red)	Schmincke AEROCOLOR
	S350(2)	28 350	Magentarot (magenta red)	Schmincke AEROCOLOR
**violet**	S410	28 410	Purpurviolett (purple-violet)	Schmincke AEROCOLOR
	S416	28 416	Tiefviolett (deep violet)	Schmincke AEROCOLOR
	RACBV		Blau-violett (blue-violet)	Rotring ArtiColor
**green**	S510	28 510	Türkisgrün (turquoise-green)	Schmincke AEROCOLOR
	S520	28 520	Zinnobergrün (cinnabar green)	Schmincke AEROCOLOR
	S540(1)	28 540	Grün (green)	Schmincke AEROCOLOR
	S540(2)	28 540	Grün (green)	Schmincke AEROCOLOR
	S566	28 566	Maisgrün (maize green)	Schmincke AEROCOLOR
	S570	28 570	Gelbgrün (yellow-green)	Schmincke AEROCOLOR
	RACBG		Blau-grün (blue-green)	Rotring ArtiColor
	RACG		Grün (green)	Rotring ArtiColor
	RACPG	R 597 418	Saftgrün (sap green)	Rotring A.C.P.
**blue**	WNPC		Process cyan	Winsor & Newton (W&N) Liquid Acrylic Color
**brown**	RACPB	R 597 425	Lichter Ocker, natur (light ochre, natural)	Rotring A.C.P.

#### 2.1.2 Acrylic Ink Mock-Ups on Watercolor Paper

Four mock-up series (I–IV, Figure 1) were created, each featuring the same six inks applied to watercolor paper. Among these, two were acrylic inks: one with the code RACGY (*Goldgelb*, Rotring ArtiColor) and the other with the code S324(3) (*Zinnoberrot*, Schmincke AEROCOLOR). These acrylic inks are the focus of the present study (Table 1). Their selection was based on preliminary MFT results on the acrylic ink mock-ups on Japanese paper, suggesting high light stability. The aim was to confirm their stability under actual museum conditions (45–55% relative humidity, 18–24 °C, 50 Lux). The remaining four inks depicted in Figure 1 are watercolor dye-based inks, which behave differently, and are discussed in a separate, unpublished study.

**Figure 1:**
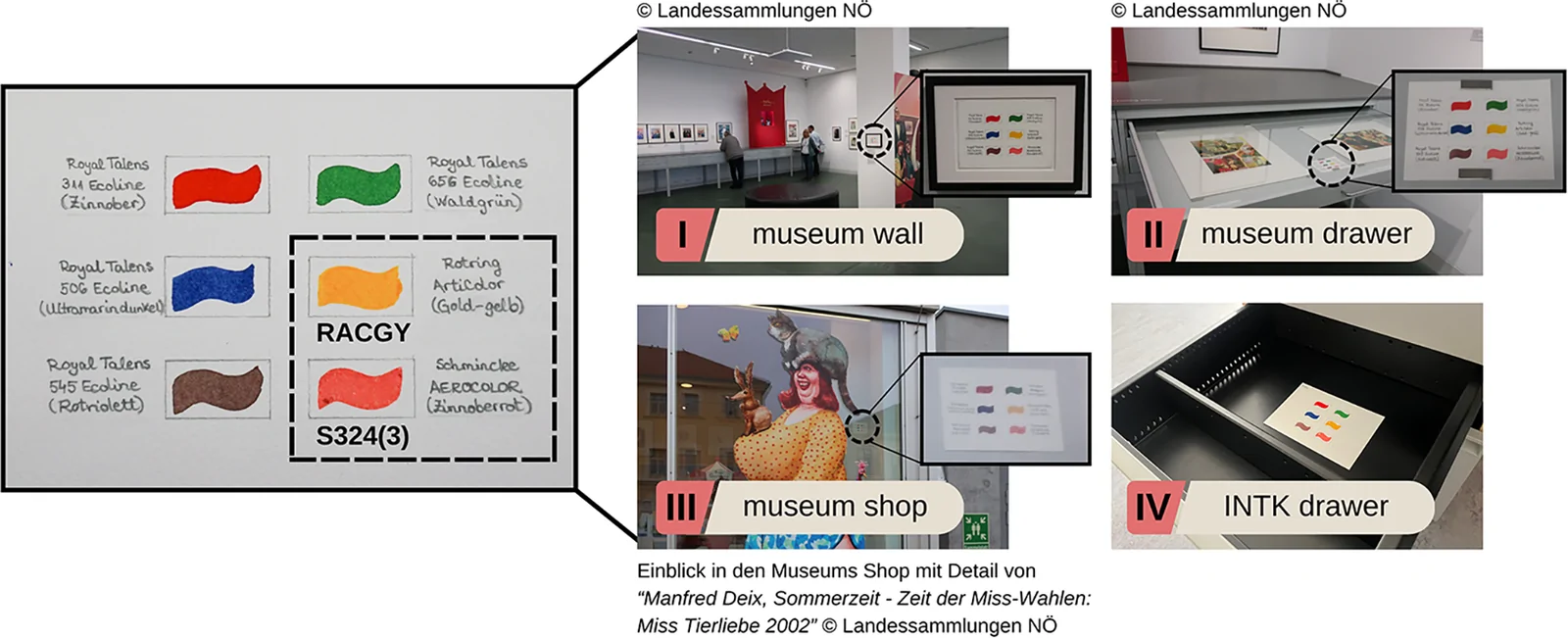
Four acrylic ink mock-up series (I–IV) on watercolor paper displayed for one year in the *I LOVE DEIX* exhibition (17 February 2024–02 February 2025) at the Karikaturmuseum Krems, Austria; images I–III © Landessammlungen NÖ. The series includes six inks, of which only two are acrylic and the focus of this study: RACGY (yellow, Rotring ArtiColor) and S324(3) (red, Schmincke AEROCOLOR). The mock-up series were displayed as follows: series I: on the museum wall; series II: in a museum drawer; series III: in the museum shop window with exposure to daylight; and series IV: in a drawer at our institute (INTK). Series I and II were displayed in the exhibition. Series III and IV served as aging references.

### 2.2 Aging of Acrylic Ink Mock-Ups and Display in the Museum

#### 2.2.1 Artificial Aging Using the Microfading Tester (MFT)

Artificial aging using MFT with an LED light source was applied only to acrylic ink mock-ups on Japanese paper at the Landessammlungen NÖ. MFT was selected to simulate LED indoor lighting, as frequently used in museum displays. Since MFT produces only microfaded spots (∼0.2 mm^2^), a larger surface area (2 mm^2^) was irradiated to ensure enough sample material for subsequent μ-Raman and MeV SIMS analyses. Each larger area consisted of 16 microfaded/measurement spots, each exposed to light for 10 min. To ensure precise positioning of each aging/analysis point, the margins of the target area were marked in pencil with small tick marks corresponding to each measurement spot. The mock-ups were then repositioned under the MFT between successive points, using these marks to align each spot consistently.

The MFT device (Instytut Fotonowy, Poland) is equipped with an LED light source (2733 K) with a spectral range of 400–750 nm, delivering a maximum output of 4 mW at the focal point. Data were acquired using a *half-light* current of 50 mA and a *full-light* current of 700 mA, with a vertical step size of 0.05 mm and a spotlight diameter of 0.5 mm at the focal point. The cumulative light dose used in this work was 1.3 Mlux·h. For context, a one-year exhibition at 50 lux (8 h/day, 6 days/week) of works on paper, generally regarded as highly light-sensitive, results in a total annual exposure of ∼0.12 Mlux·h, according to the museum lighting standard CIE157:2004 still in use today.^39^ Thus, the MFT approach employed here provided a light dose roughly ten times greater than a standard exhibition cycle. Color differences (Δ*E*_00_, most related to human color perception)^40,41^ were continuously recorded during increasing LED light exposure at a rate of 0.002 Mlux·h/s. Prior to measurements, the instrument was calibrated using a white reference (BaSO_4_, Instytut Fotonowy, Poland). Illuminance at the tested spots was not measured throughout the analyses, but the reported light dose reflects the most recent manufacturer calibration, performed shortly before the measurements.

#### 2.2.2 Display under Actual Museum Conditions

To assess the effects of museum light exposure over one year on SOPs in acrylic inks, the ink mock-ups on watercolor paper were displayed during the “*I LOVE DEIX”* exhibition (17 February 2024–02 February 2025) at the Karikaturmuseum Krems, Austria. The exhibition conditions adhered to current conservation guidelines for works on paper,^42^ with continuous climate monitoring (45–55% relative humidity, 18–24 °C, 50 Lux).

The mock-up series I–IV were displayed as follows (Figure 1):

- Series I: mounted on a wall in a windowless exhibition room and framed with a Mirogard glass (82% UV protection), receiving the same light exposure as the original caricatures.
- Series II: stored in a drawer together with the light-sensitive caricatures in the same windowless exhibition room as Series I; light exposure was limited and dependent on drawer opening/closing by visitors during viewing.
- Series III: placed in the museum shop window and exposed to sunlight.
- Series IV: kept in a drawer at the INTK under dark conditions without opening.

### 2.3 Real Sample from the Caricature “Tanzkür 1999 auf dem Eis” of Erich Sokol

The analytical protocol initially developed on ink mock-ups for identifying SOPs was applied to the real sample taken from “*Tanzkür 1999 auf dem Eis*” (1999, Landessammlungen NÖ), a caricature by Erich Sokol. To preserve the artwork’s integrity, curators strictly limited sampling to the outer margin of the caricature, allowing only the blue-colored area to be sampled. The sample, showing thin, diluted application of blue ink, consisted of blue acrylic ink on paper fibers. It was selected for its presumed low pigment content to assess the sensitivity and reliability of the protocol, as many acrylic ink artworks are known for their translucent layers that resemble watercolors.

### 2.4 μ-Raman Spectroscopy

Dispersive μ-Raman spectroscopy was performed using an inVia QONTOR confocal Raman microscope (Renishaw, UK). Two excitation sources were employed: a 532 nm Nd:Yag laser (100 mW) with a 2400 l/mm grating, and a 785 nm diode laser (300 mW) with a 1200 l/mm grating. Spectra were collected using a 50× long working distance (LWD) objective and a CCD detector (1024 × 254 pixels, Peltier-cooled to −70 °C). Wavenumber calibration was performed using a silicon (Si) standard, ensuring the υ_1_ band of Si at 520.7 ± 0.5 cm^–1^.

Twelve spots were analyzed for each acrylic ink mock-up: three on unaged and three on aged ink areas for each excitation wavelength (532 and 785 nm). For the 532 nm laser, spectra acquisition was performed using a 15 s exposure time, 3 accumulations, and 0.5 mW laser power at the sample. For the 785 nm laser, spectra acquisition was performed using a 15 s exposure time, 5 accumulations, and a laser power of 1.1 mW at the sample. Acquisition parameters were adjusted, when necessary, based on SOP Raman signal intensity. All spectra were acquired and evaluated using the WiRE software (v.5.6., Renishaw, UK). For SOP identification in measured inks, spectra were compared with the INTK’s internal materials Raman database and Raman spectra libraries SOPRANO,^43^ allowing identification of up to two SOPs per ink. The extended statistical data evaluation of Raman spectra to better track differences among the spectra is detailed in Subsection 2.9.

### 2.5 MeV SIMS

For MeV SIMS analyses, small samples from all acrylic ink mock-ups and the real sample were removed with a scalpel, including the ink and the paper substrate. Subsequently, samples were pressed into pure indium (in teardrops, ≥99.9%, Sigma-Aldrich, USA) using a flat aluminum plate. MeV SIMS measurements were performed using the linear time-of-flight (TOF) mass spectrometer installed at the heavy-ion microprobe at the Ruđer Bošković Institute (Croatia) using the setup described in Tadić et al.^44^ The 8 MeV Si^4+^ primary ion beam was generated by a 6.0 MV EN Tandem Van de Graaff accelerator. The beam was focused to a spot size of 10 × 10 μm^2^ and rastered over areas in the range of 450 × 450 μm^2^ to 750 × 750 μm^2^. Each sample was measured in 2–3 positions, depending on the fragment and scan size. Measurements were conducted in positive-ion mode under high vacuum (10^–6^ to 10^–7^ mbar), with the target potential set at +5 kV and the Microchannel Plate (MCP) at −2 kV. All experimental and acquisition parameters were controlled with the in-house SPECTOR software package^45^ and processed in the *mMass*-Open Source Mass Spectrometry Tool (version 5.5.0).^46^ Base peak normalization with baseline correction was employed for spectra comparison before and after aging.

The resulting MeV SIMS spectra were calibrated individually to account for differences in sample geometry that affect the arrival time of the secondary ions.^47^ To ensure consistent calibration, a common peak list was applied to all spectra.^48^ Five ions were selected: H^+^, H_2_^+^, Na^+^, C_2_H_5_^+^, and C_8_H_5_O_3_^+^, as they were present in each spectrum. In addition, molecular ions or protonated molecules of SOPs were included to extend the calibration across a wider mass range.

The internal MeV SIMS SOP library, built during this work and our previous joint projects using the same MeV SIMS setup, was employed for pigment identification.^25,34^ The library covered phthalocyanine, monoazo, naphthol AS, β-naphthol, anthraquinone, quinacridone, diketopyrrolo-pyrrole, and triarylcarbonium pigments, but lacked diarylide yellows, dioxazine, perinones, and several other naphthol AS pigments. To address this, pure pigment powders chosen from these classes were analyzed alongside acrylic ink mock-ups (Table S1, SI). The resulting MeV SIMS reference spectra were evaluated by comparison with DTMS^49^ and LDI-MS^50^ on the same SOPs. Reference spectra and their interpretations are presented in Figures S3–S10 and Table S2 of the SI. The chemical structures of the pigments and their fragment ions were illustrated using ChemSketch (freeware, v.2023.2.4).

### 2.6 Pyrolysis–Gas Chromatography/Mass Spectrometry (Py-GC/MS)

As this study focused on SOP identification and photodegradation, only selected acrylic inks were analyzed by Py-GC/MS, since this technique is not effective for identifying SOPs from all chemical classes (see Section 1). However, to identify binders and additives and observe formulation differences, a representative ink from each trademark (Schmincke AEROCOLOR, Rotring ArtiColor, Rotring A.C.P., and Winsor & Newton) was chosen for Py-GC/MS analysis. Specifically, inks S510 (green), RACBG (green), RACPG (green), and WNPC (blue) were analyzed because they all contain phthalocyanines (PB15:1 and/or PG7). As this was the only pigment class present in all four studied trademarks, selecting these inks ensured comparable pigment-related pyrolyzates and minimized interference with binders and additives identification. Samples were taken from the mock-ups on Japanese paper, which is a filler-free substrate that does not affect results.

Py-GC/MS was performed in single-shot mode (flash pyrolysis), a technique well-established for analysis of acrylic emulsion paints.^28,29,51,52^ Analyses were performed using a GCMS-QP2020 NX instrument (Shimadzu, Japan) coupled with an EGA/Py-3030D multi-shot pyrolyzer (Frontier Laboratories, Japan). A 5 % diphenyl polydimethylsiloxane capillary column (30 m length × 0.25 mm ID, and 0.25 μm film thickness, UA5, Frontier Laboratories, Japan) was employed. The parameters were adapted from Fardi et al.^52^ on acrylic emulsion paints (Table S3, SI). 500 μg of each sample (ink on paper fibers) was taken using a scalpel, placed into 50 μl sample cups (Eco-Cup LF, Frontier Laboratories, Japan), and analyzed directly.

Data evaluation was carried out using AMDIS (ver. 2.71, NIST) software. The obtained results were processed in the Expert System for Characterization with AMDIS Plus Excel (ESCAPE), developed at the Getty Conservation Institute (Los Angeles, USA),^53^ for semiautomatic compound identification. Unidentified compounds were assigned by examining the mass spectra of individual chromatographic peaks using the NIST/EPA/NIH Mass Spectral Library 2023.

### 2.7 VIS Reflectance Spectroscopy

To record the VIS reflectance spectra and possible color differences (Δ*E*_00_) of the acrylic ink mock-ups on watercolor paper displayed under actual museum conditions, an X-Rite eXact Basic Spectrophotometer (X-Rite, USA) was used. Measurements were performed directly on the mock-ups under “M1 conditions”, simulating daylight (illuminant D50, standard observer 2°). Spectral data were acquired at 10 nm intervals over the 400–700 nm range using a 4 mm aperture.

### 2.8 Optical Microscopy

Optical microscopy was used to examine whether MFT and exhibition in the museum induced any visible changes under magnification, such as binder loss from the surface or fewer pigment agglomerates. A VHX-6000 digital microscope (Keyence, Belgium) equipped with a 5700 K LED light source and an LCD monitor was employed for this purpose. Images were acquired using a VH-Z100R objective (100× – 1000× magnification) and a CMOS camera with a 1/1.8-inch sensor, providing a pixel resolution of 1600 × 1200.

### 2.9 Statistical Data Evaluation

Multivariate analysis (MVA) was carried out alongside the usual qualitative Raman spectral analysis to rapidly classify acrylic inks by color, identify pigment mixtures, and indicate aging-related changes within a single model. This approach enabled a clear comparison among inks of the same color.

MVA was performed solely on the Raman datasets, as μ-Raman enabled the acquisition of multiple spectra per mock-up, sufficient for constructing a robust statistical model. The limited number of MeV SIMS spectra per sample due to restricted accelerator beam time slots prevented statistical evaluation.

The MVA workflow (Figure 2) was adapted from Morais et al.^54^ and was performed in RStudio (v.2025.05.1), using raw (unprocessed) spectra with categorical variables: *Sample* (sample name), *Color* (as defined in Table 1), and *Aging* (unaged/aged). Separate models were built for the 532 and 785 nm laser Raman datasets collected from unaged and MFT-aged acrylic ink mock-ups on Japanese paper.

**Figure 2:**
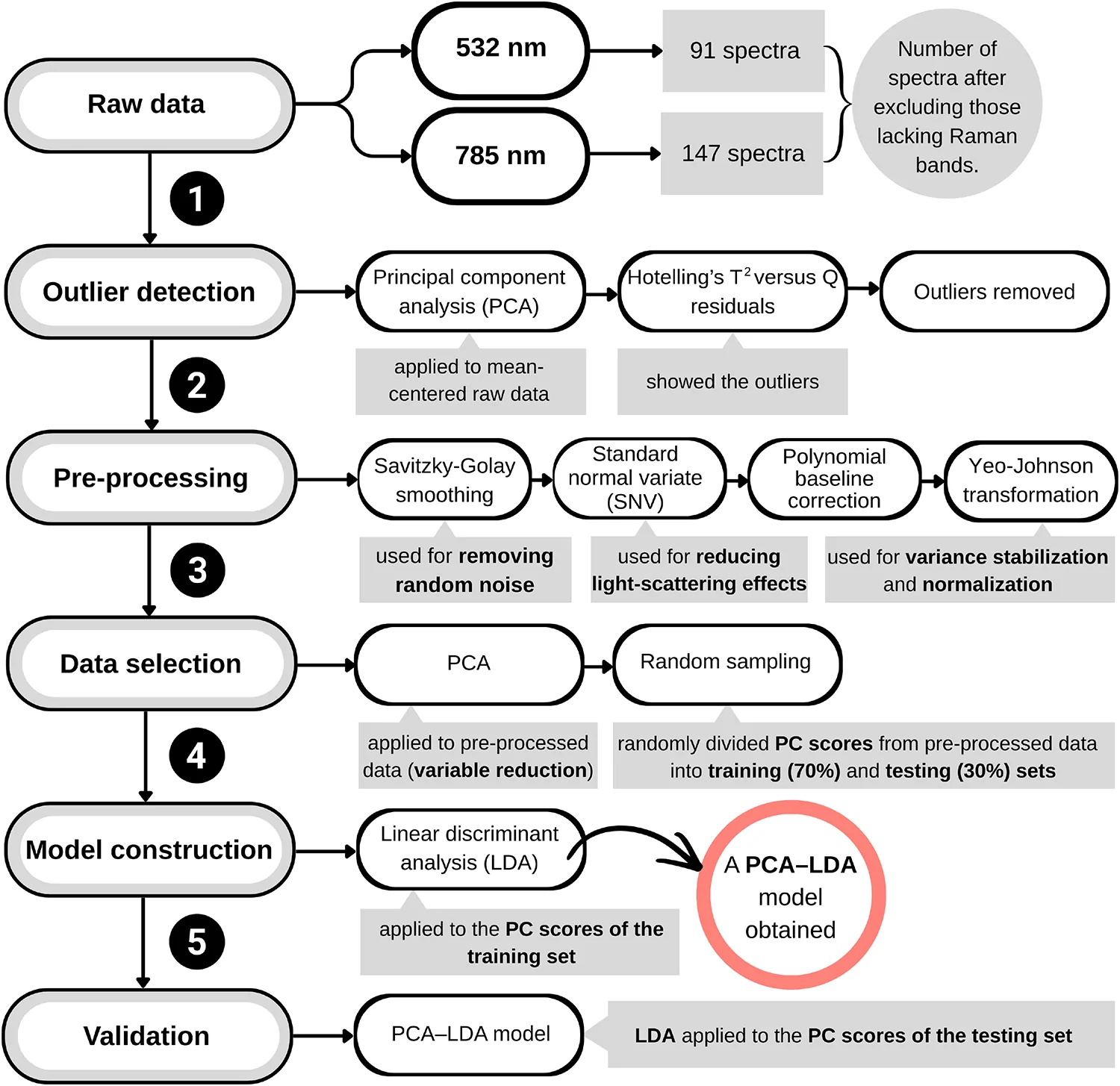
Workflow of the multivariate analysis applied to the 532 and 785 nm laser Raman datasets collected from unaged and aged acrylic ink mock-ups.

Linear discriminant analysis (LDA), a supervised method for classifying objects based on predefined classes,^55^ was employed. However, LDA is sensitive to unequal variance structures among classes and performs poorly when the number of variables exceeds the number of samples.^54,55^ To overcome this, LDA was applied to PC scores from the principal component analysis (PCA) of pre-processed data. PCA was applied to reduce collinearity and minimize overfitting.^56^ Only PCs explaining over 95% of the cumulative variance were used. Prior to classification, multivariate analysis of variance (MANOVA) was performed to assess the influence of the variable *Aging*. Since *Aging* was binary, only one linear discriminant (LD) could be generated, preventing a multidimensional model. Therefore, variable *Color* was used, as it contained seven predefined color classes. Model quality was evaluated through accuracy, sensitivity, and specificity, which measure overall predictive performance, the ability to correctly identify inks of a given color, and the ability to exclude inks from other classes, respectively.

The PCA–LDA models developed from the 532 and 785 nm Raman datasets (ink mock-ups on Japanese paper) were subsequently applied to the dataset of the mock-ups on watercolor paper displayed under museum conditions, as well as to the real sample. All corresponding Raman spectra were processed using the established workflow depicted in Figure 2, and the resulting PC scores were included in the testing sets of the original Raman 532 and 785 nm laser models.

## 3 Results and Discussion

### 3.1 Acrylic Ink Mock-Ups on Japanese Paper

The following subsections present the results for the acrylic ink mock-ups on Japanese paper. Subsection 3.1.1. focuses on SOP identification and stability after MFT aging through a combination of MeV SIMS and μ-Raman, the latter supported by MVA analysis. In Subsection 3.1.2. results on identification of binders and additives by Py-GC/MS are presented.

#### 3.1.1 Synthetic Organic Pigments (SOPs)

SOP identification and stability assessed by MeV SIMS are discussed by ink color in the following order: yellow, red, magenta, violet, green, blue, and brown. Since μ-Raman identification of SOPs is well-established in the literature,^22,43,57,58^ qualitative Raman results are summarized alongside MeV SIMS data rather than described in detail. Following this, the PCA–LDA models constructed from the 532 and 785 nm Raman datasets are presented.

In yellow inks S230, RACGY, and RACY, diarylide and monoazo yellows were determined. PY83 was identified in S230 and RACGY by MeV SIMS via its [M]^+^ ion at *m/z* 816/818, and fragment ions [C_8_H_10_ClNO_2_]^+^, [C_12_H_10_Cl_2_N_2_]^+^, and [C_24_H_21_Cl_3_N_4_O_4_]^+^ at *m/z* 187/189, 252/254, and 534/536, respectively. [C_12_H_10_Cl_2_N_2_]^+^, arising from azo-bond cleavage, is a marker of diarylide yellows and appeared in all reference spectra (SI, Figures S3 to S6). PY13, identified by its [M]^+^ ion at *m/z* 684/686 and its main fragment ion at *m/z* 468/470 (azo-bond cleavage), is also present in RACGY (Figure 3A), while in yellow RACY only PY74 (monoazo yellow, [M]^+^ ion at *m/z* 386) was detected. Regarding light stability, only RACGY showed significant changes after MFT aging: the PY13 [M]^+^ ion was no longer detected, while PY83 remained clearly visible, showing only a slight decrease in relative intensity (Figure 3A). μ-Raman confirmed the presence of PY83 in S230 and RACGY, while PY74 was found in RACY. PY13, however, was not detected in RACGY by μ-Raman.

**Figure 3:**
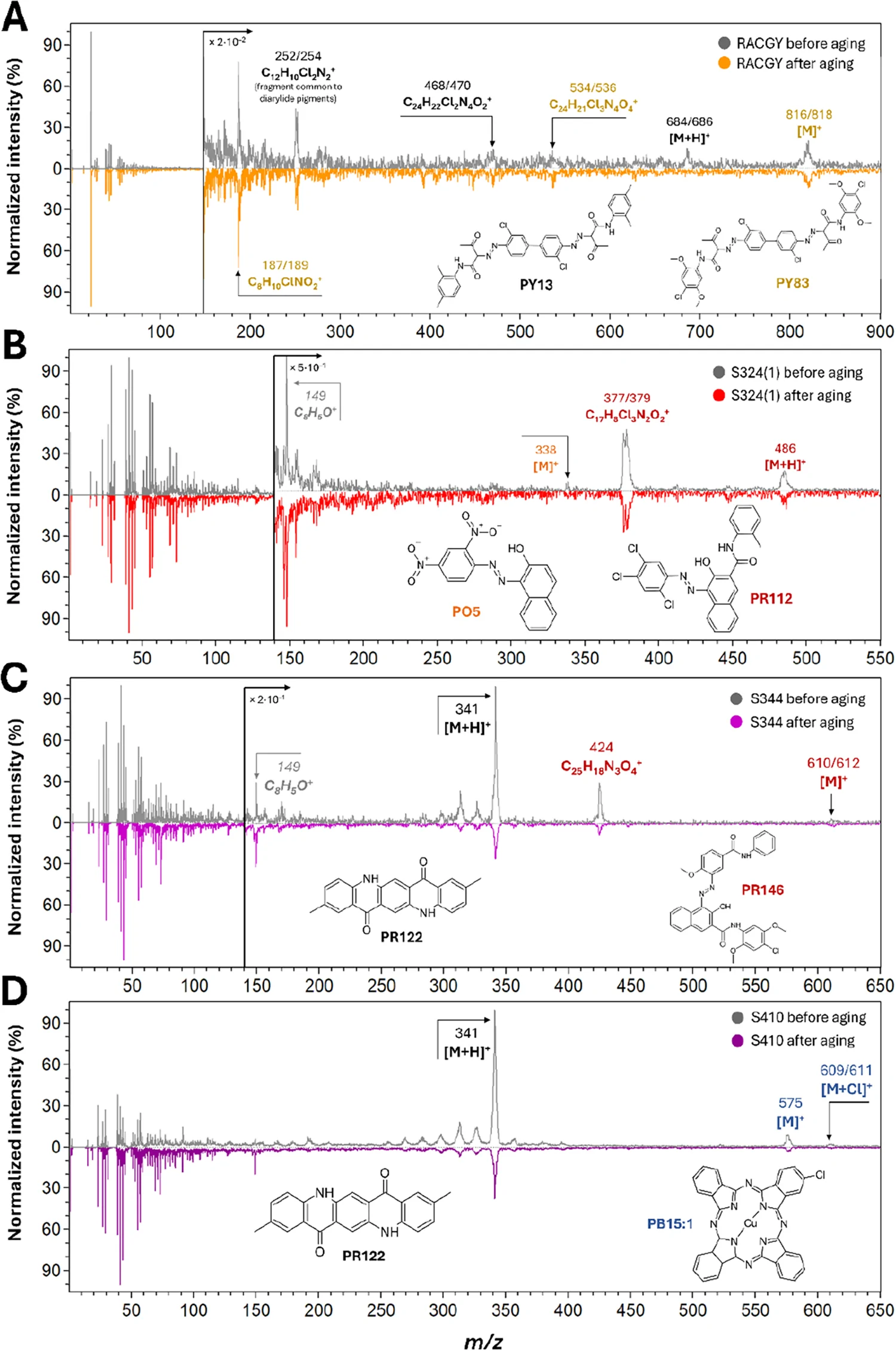
8 MeV Si^4+^ positive-ion mode MeV SIMS spectra of representative inks from four different color classes, acquired before and after artificial photoaging using microfading tester (MFT): (A) RACGY (yellow, *Goldgelb*, Rotring ArtiColor), (B) S324(1) (red, *Zinnoberrot*, Schmincke AEROCOLOR), (C) S344 (magenta, *Krapprosa*, Schmincke AEROCOLOR), and (D) S410 (violet, *Purpurviolett*, Schmincke AEROCOLOR).

In the red inks S324(1–3), S330, and RACR, naphthol AS pigments were detected, either alone or mixed with monoazo or perinone pigments. PR112 was identified in all S324(1–3) and S330 inks via its [M + H]^+^ ion at *m/z* 486 and a main fragment ion [C_17_H_8_Cl_3_N_2_O_2_]^+^ at *m/z* 377/379 (amide bond cleavage). In S324(1), PR112 co-occurs with the monoazo pigment PO5 ([M]^+^ ion at *m/z* 338), while S324(2–3) likely contain the perinone pigment PO43 ([M + H]^+^ ion at *m/z* 413; reference spectrum in Figure S7, SI). Although the [M]^+^ ion of PO5 had a low intensity (Figure 3B), it was clearly identified by μ-Raman. PO43 (trans isomer) was indistinguishable from PR194 (cis isomer).^59^ Schmincke currently lists PO43, not PR194, in its AEROCOLOR trademark,^60^ and Herbst et al.^59^ note that despite its higher cost, PO43 is favored for its superior light stability. PO5 in S324(1) was therefore likely replaced by PO43 in later formulations to improve lightfastness, given PO5’s known instability.^59^ Red ink S330 contains only PR112, whereas RACR contains PR7 (reference spectrum in Figure S8, SI), another naphthol AS pigment identified by its [M]^+^ ion at *m/z* 463/465 and a main fragment ion [C_18_H_12_ClN_2_O_2_]^+^ at *m/z* 323/325 (amide bond cleavage). SOP-related peak intensities decreased following MFT aging, most notably in S324(1), where the low-intensity PO5 [M]^+^ ion became undetectable. For all red inks, only μ-Raman with 785 nm laser excitation was quite effective, as naphthol AS pigments exhibited strong fluorescence under 532 nm excitation.

In all magenta inks, S340(1–2), S344, and S350(1–2), either PR146 (naphthol AS), PR122 (quinacridone), or mixtures of both were identified. In S340(1) and S340(2), PR146 was determined via its [M]^+^ ion at *m/z* 610/612 and the main fragment ion at *m/z* 424 [C_25_H_18_N_3_O_4_]^+^ from amide bond cleavage (reference spectrum in Figure S9, SI). S350(1) and S350(2) showed no differences, both containing only PR122 ([M + H]^+^ at *m/z* 341). S344 contains both PR146 and PR122 (Figure 3C). Following MFT aging, no spectral changes were observed in the magenta inks. μ-Raman identified each pigment individually but not in mixtures (e.g., S344), as PR122 showed strong fluorescence characteristic of quinacridones,^61^ attributed to their rigid π-conjugated structure. Thus, only PR146 was detected in S344, and strictly under 785 nm excitation.

Three violet inks were analyzed: S410, S416, and RACBV. They contain PB15:1 (phthalocyanine) with PR122 (quinacridone), PB15:1 with PV23 (dioxazine), or PV23 alone. PB15:1 (chlorine-containing phthalocyanine) was detected in S410 and S416 via its [M]^+^ and [M + Cl]^+^ ions at *m/z* 575 and 609/611. In S410, PR122 was additionally identified by its [M + H]^+^ ion at *m/z* 341 (Figure 3D) and [2M]^+^ at *m/z* 680. In S416, PV23 was identified by its [M]^+^ ion at *m/z* 588/590 (reference spectrum in Figure S10, SI). In RACBV, only PV23 was determined. Consistent with previous studies,^34^ μ-Raman confirmed only the presence of a PB15:x polymorph in S410, while MeV SIMS determined the exact polymorph (PB15:1). However, μ-Raman identified PV23 in S416 and RACBV.

In green acrylic inks (S510, S520, S540(1–2), S566, S570, RACBG, RACG, and RACPG) PG7 or PG36 (both phthalocyanines) were found alone or mixed with PB15:1 (phthalocyanine) or PY74 (monoazo) and PY14 (diarylide yellow). The only exception was green RACPG, consisting of PB15:x and PY97 (monoazo). PG7 was identified in S510, S520, RACBG, and RACG inks via its characteristic series of molecular ions with 12–16 Cl substituents on the phthalocyanine ring. PG36 was found in S540(1–2), S566, and S570 via molecular ions reflecting different Br/Cl substituent ratios. PB15:1 was identified in S510 and RACBG, whereas RACPG showed a PB15:x polymorph with PY97 ([M]^+^ ion at *m/z* 590/592). S566 additionally contained PY74 ([M]^+^ ion at *m/z* 386) and PY14 ([M]^+^ ion at *m/z* 656/658; Figure 4A), likely added to lighten the hue. Only S566 presented significant spectral changes after MFT aging, as PY14 became undetectable, likely due to its low concentration (Figure 4A). μ-Raman identified only PG7 and PG36.

**Figure 4:**
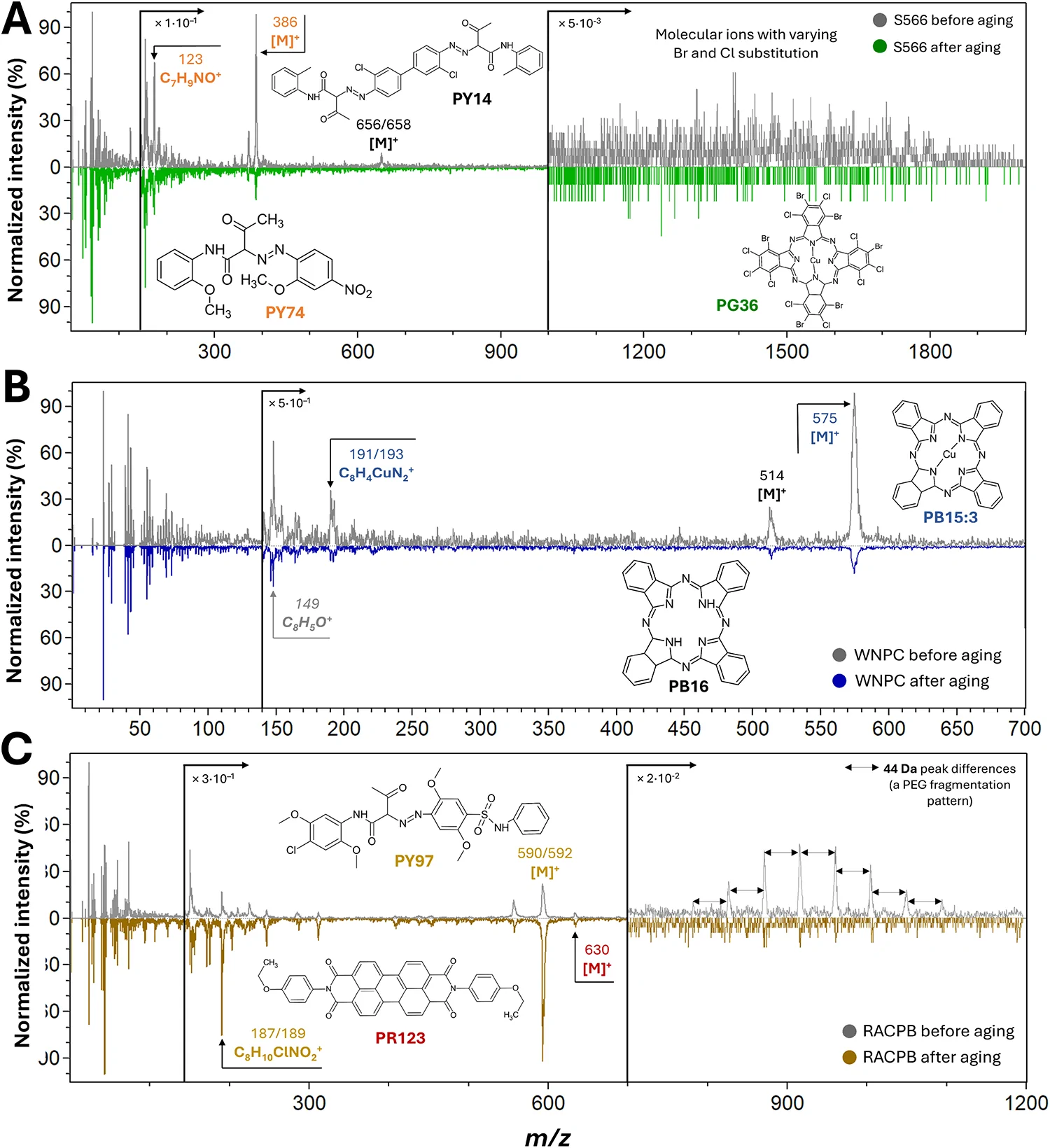
8 MeV Si^4+^ positive-ion mode MeV SIMS spectra of representative ink from three different color classes, acquired before and after artificial photoaging using a microfading tester (MFT): (A) S566 (green, *Maisgrün*, Schmincke AEROCOLOR), (B) WNPC (blue, W&N), and (C) RACPB (brown, *Lichter Ocker, nature*, Rotring A.C.P.), all of which contained mixtures of SOPs.

In the blue ink WNPC, a mixture of PB15:3 and PB16 (Figure 4B) was determined. The [M]^+^ ion of PB15:x (Cu-phthalocyanine) at *m/z* 575 and fragment ion [C_8_H_4_CuN_2_]^+^ at *m/z* 191/193, along with the absence of [M + Cl]^+^ (*m/z* 609/611), confirmed the PB15:3 polymorph. This is consistent with WNPC’s name (*Process Cyan*), as PB15:3 is typically used for cyan in printing inks due to its greenish-blue hue.^62^ Furthermore, metal-free PB16 was identified via its [M]^+^ ion at *m/z* 514. μ-Raman showed the presence of a polymorph of PB15:x and PB16.

The brown ink RACPB contains a mixture of PR123 (perylene) and PY97 (monoazo) (Figure 4C). PR123 was identified by its [M]^+^ ion at *m/z* 630, and PY97 by its [M]^+^ ion at *m/z* 590/592 and a dechlorinated fragment ion at *m/z* 555 ([M – Cl]^+^). μ-Raman could not distinguish PR123 from PR190 (both perylenes), as their spectra are nearly identical; the two SOPs differ only by two additional methylene groups (−CH_2_−) in PR123. MeV SIMS confirmed the presence of PR123, used for its bright medium-red hue.^59^ For this ink, SOP molecular and fragment ion intensities increased after MFT aging, likely due to the disappearance of PEG (a nonionic surfactant), from the ink surface (Figure 4C; see Subsection 3.1.2. *for additive discussion*). This facilitated detection of underlying pigment grains via increased surface presence.

Unaged acrylic ink-on-paper mock-ups allowed clear detection of SOP molecular ions, protonated molecules, and characteristic fragment ions, unlike our previous MeV SIMS study on acrylic emulsion paints analyzed directly on a substrate, which yielded limited molecular information.^35,63^ In acrylic emulsion paints, SOP detection is hindered by pigment particle encapsulation within the polymer matrix and the formation of a “medium skin”, a nanometer-thick binder layer at the film surface, which is particularly relevant given MeV SIMS’s nanometer-range surface sensitivity. Accessing these pigments typically requires partial binder removal via UV aging or by cutting and pressing a sample into indium.^35,63^ In contrast, acrylic inks contain smaller pigment particles and are less viscous, preventing formation of a continuous medium skin. Thus, pigment particles remain exposed and readily accessible at the paper surface, enabling direct detection by MeV SIMS.

After MFT aging, SOP [M]^+^ ions were still detected by MeV SIMS (Figures 3 and 4); however, relative peak intensities decreased in most cases, sometimes by up to an order of magnitude. While this decrease could reflect photodegradation of the SOPs, an alternative explanation must be considered: MFT aging appeared to promote surfactant migration to the surface in Rotring ArtiColor inks (RACG, RACBV, and RACY) and two Schmincke AEROCOLOR inks (S324(3) and S540(2)), a phenomenon previously observed in acrylic emulsion paints aged under fluorescent light.^64^ MeV SIMS detected surfactant-related peaks at *m/z* 495, 523, and 551, the latter attributed to the [M – Cl]^+^ of dimethyldioctadecylammonium chloride (C_38_H_80_ClN) (Figure S11, SI).^65–67^ Although cationic surfactants are typically incompatible with anionic ones, they can coexist with the nonionic and amphoteric surfactants^68^ identified here by Py-GC/MS (detailed in SI). Though rarely used in acrylic emulsion paints, where nonionic–anionic surfactant mixtures prevail,^69^ their presence in acrylic inks may relate to their affinity for negatively charged paper surfaces.^66^ This surfactant migration suggests that, in these inks, surfactants or other additives may progressively cover the pigment particles at the surface after MFT aging, becoming more prominent in the MeV SIMS spectra relative to SOPs. Since MeV SIMS probes only the outermost surface layer, such an overlayer effect would reduce SOP secondary ion yield without any underlying chemical change to the pigments themselves.

Overall, the combination of MeV SIMS and μ-Raman proved highly effective for identifying SOPs in acrylic inks, herein comprising pigments from eight chemical classes. MeV SIMS results are summarized in Table 2, and Figure 5 presents the identified SOPs alongside the most effective combinations of MeV SIMS and Raman, including the Raman laser used.

**Figure 5:**
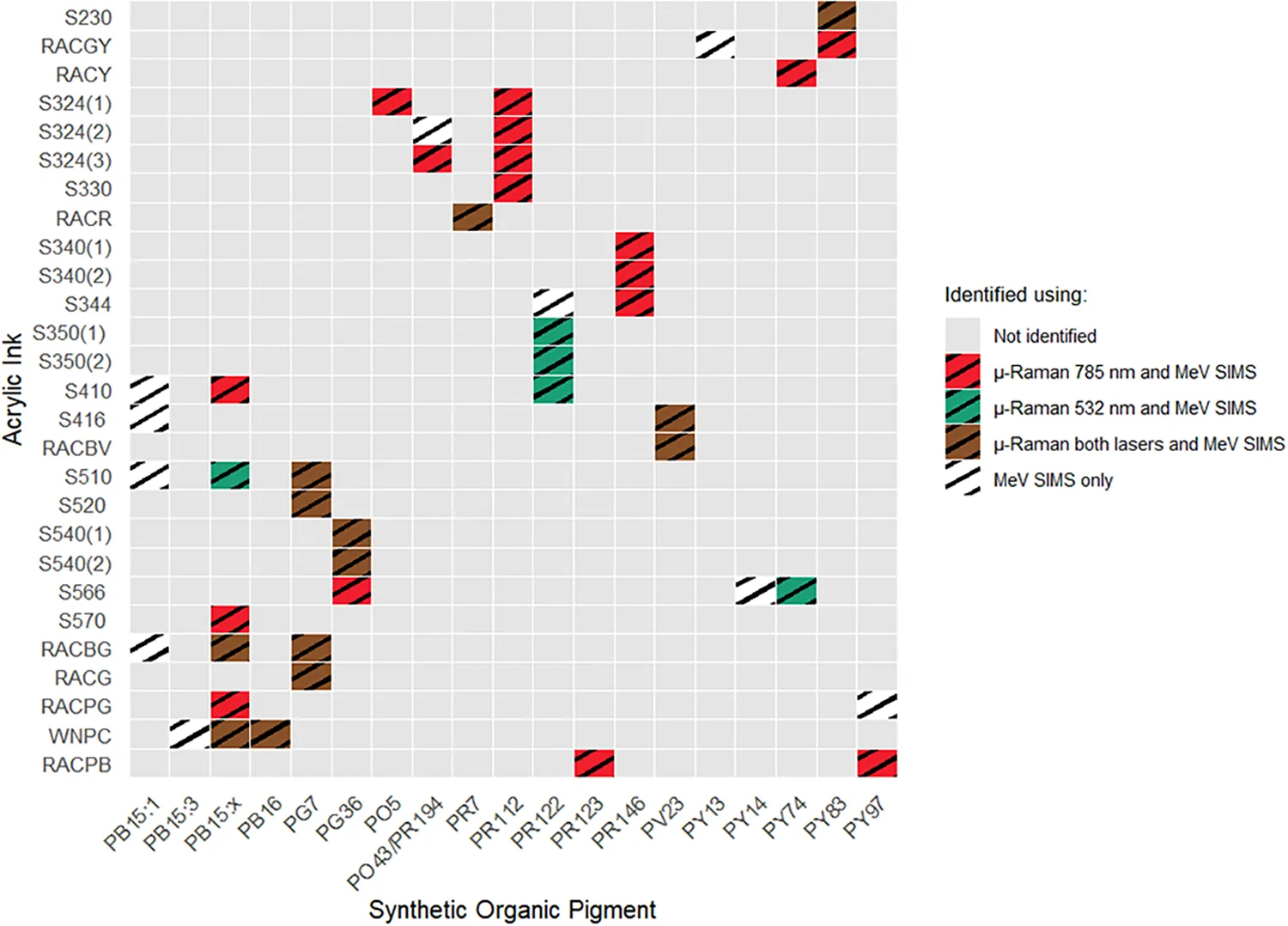
Summary of SOPs identified in the acrylic inks using MeV SIMS and/or μ-Raman (532 nm and/or 785 nm laser). Each row represents one acrylic ink, and each column corresponds to the specific SOP identified. Colored cells indicate the analytical techniques that successfully detected each pigment. Acrylic inks are listed along the *y*-axis from top to bottom in the same order as in Table 2.

**Table 2:** Synthetic Organic Pigments Identified by MeV SIMS, with Their Main Corresponding Peaks^a^

**general assigned color**	**acrylic ink on Japanese paper**	**pigment(s) identified**	**chemical class**	**pigment-related peaks** ***(m/z)***
**yellow**	S230	PY83	diarylide	172/174,187/189, 252/254, 534/536, 816/818
	RACGY	PY83	diarylide	172/174, 187/189, 252/254, 534/536, 816/818
		PY13	diarylide	468/470, 684/686
	RACY	PY74	monoazo	123, 168, **386**
**red**	S324(1)	PR112	naphthol AS	115, 128, 142, 156, 169, 377/379, **486**
		PO5	monoazo	**338**
	S324(2)	PR112	naphthol AS	115, 128, 142, 156, 169, 377/379, **486**
		PO43	perinone	**413**
	S324(3)	PR112	naphthol AS	115, 128, 142, 156, 169, 377/379, **486**
		PO43	perinone	**413**
	S330	PR112	naphthol AS	106, 115, 128, 142, 156, 169, 286, 377/379, **486**
	RACR	PR7	naphthol AS	115, 128, 142, 156, 323/325, 463/465
**magenta**	S340(1)	PR146	naphthol AS	115, 128, 142, 156, 226, 424, 610/612
	S340(2)	PR146	naphthol AS	115, 128, 142, 156, 226, 424, 610/612
	S344	PR122	quinacridone	312, 326, **341**
		PR146	naphthol AS	115, 128, 142, 156, 226, 424, 610/612
	S350(1)	PR122	quinacridone	312, 326, **341, 680**
	S350(2)	PR122	quinacridone	312, 326, **341, 680**
**violet**	S410	PR122	quinacridone	312, 326, **341**
		PB15:1	phthalocyanine	191/193, **575**, 609/611
	S416	PV23	dioxazine	526/528, 554/556, 560/562, 588/590
		PB15:1	phthalocyanine	191/193, **575**, 609/611
	RACBV	PV23	dioxazine	526/528, 554/556, 560/562, 588/590
**green**	S510	PB15:1	phthalocyanine	191/193, **575**, 609/611
		PG7	phthalocyanine	989, 1057, **1127**
	S520	PG7	phthalocyanine	989, 1057, **1127**
	S540(1)	PG36	phthalocyanine	characteristic series of peaks in the range 1216–1838
	S540(2)	PG36	phthalocyanine	characteristic series of peaks in the range 1216–1838
	S566	PY74	monoazo	108, 123, 152, 168, 341, 356, 371, **386**
		PY14	diarylide	656/658
		PG36	phthalocyanine	characteristic series of peaks in the range 1216–1838
	S570	PG36	phthalocyanine	characteristic series of peaks in the range 1216–1838
	RACBG	PB15:1	phthalocyanine	191/192, **575**, 609/611
		PG7	phthalocyanine	989, 1057, **1127**
	RACG	PG7	phthalocyanine	331, 989, 1057, **1127**
	RACPG	PB15:x	phthalocyanine	191/193, **575**
		PY97	monoazo	187/189, 555, 590/592
**blue**	WNPC	PB15:3	phthalocyanine	191/193, **575**
		PB16	phthalocyanine	129, **514**
**brown**	RACPB	PY97	monoazo	187/189, 555, 590/592
		PR123	perylene	**630**

a Molecular ions/protonated molecules are shown in **bold**.

The PCA–LDA model of the 532 nm laser Raman dataset, built on the training set (Figure 6A), showed moderate performance on the testing set (Figure 6B): 64.3% accuracy, 57.3% sensitivity, and 93.5% specificity. Most green inks (S510, S520, S540(1–2), S566, RACBG, RACG), the blue ink (WNPC), and two violet inks (S416 and RACBV) were correctly classified, showing good discrimination for phthalocyanine- and/or dioxazine-containing inks. Lower performance for red inks and one violet ink resulted from overlap with magenta inks, consistent with their composition: S350(1–2) and S410 contain PR122 (quinacridone), with S410’s PB15:1 (phthalocyanine) also explaining its proximity to WNPC (blue). Red ink RACR (PR7, naphthol AS), clustered near PR122-based inks. Spectral comparison confirmed a weak PR122 contribution in RACR, previously masked by low signal intensity under 532 nm excitation. Additional misclassifications involved green inks S566 and RACBG, assigned to yellow and blue clusters, respectively, due to dominant PY74/PY14 or PB15:1 contributions.

**Figure 6:**
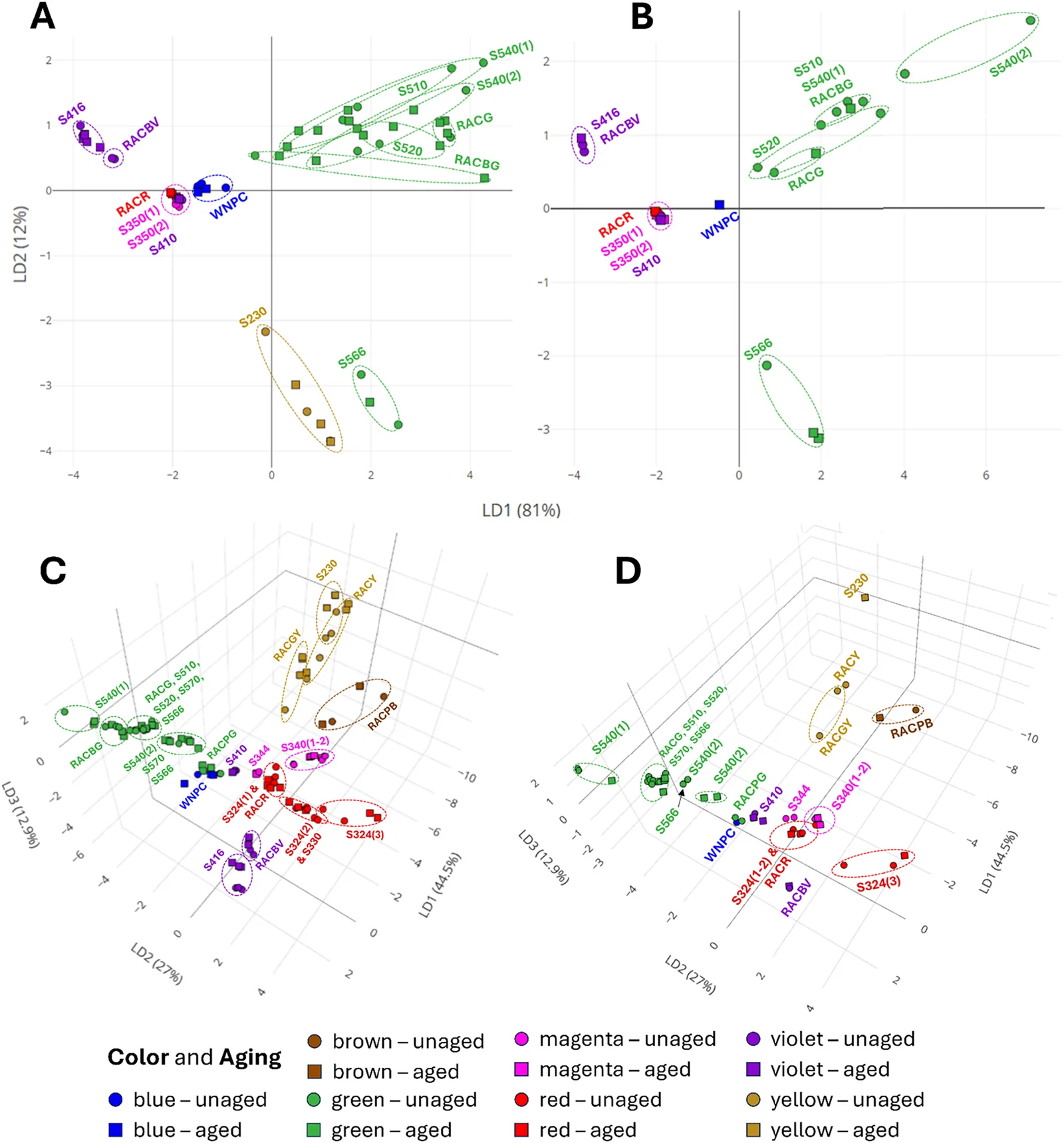
PCA–LDA models for the 532 and 785 nm Raman datasets of unaged and MFT-aged acrylic ink mock-ups on Japanese paper. Data are divided into training (70%; (A) for 532 nm, (C) for 785 nm laser) and testing sets (30%; (B) for 532 nm, (D) for 785 nm laser). Seven color clusters are defined by the *Color* explanatory variable, with individual inks marked by ellipses and labels. The shape of the data points indicates *Aging*: circles (unaged), and squares (MFT-aged).

MANOVA showed no significant MFT aging effect on PC scores (Wilks’ Lambda = 0.9905), likely because 532 nm μ-Raman predominantly detected light-stable SOPs. Despite moderate accuracy, the model confirmed minimal aging effects and revealed an additional colorant (PR122 in RACR).

The PCA–LDA model for the 785 nm laser Raman dataset built on the training set (Figure 6C) performed significantly better than the 532 nm model on the testing set (Figure 6D): 86.7% accuracy, 87.0% sensitivity, and 97.0% specificity. Sensitivity was near 100% for most color classes except violet (40%), with only three misclassifications: S410 (violet), RACPG (green), and S344 (magenta), all attributable to SOP mixtures. Unlike the 532 nm model, RACR was correctly classified as red, since the red pigment PR7 was identified in the Raman spectra under 785 nm excitation. Green ink S566 was likewise correctly classified as green despite containing yellow pigments. This demonstrates the advantage of 785 nm excitation for identifying and discriminating SOP combinations in the inks studied in this work.

MANOVA indicated a significant MFT aging effect on PC score variance for the 785 nm dataset (Wilks’ Lambda = 0.0052), unlike at 532 nm, most evident in yellow and red inks. For example, yellow ink RACGY (containing PY83 and PY13) shifted toward yellow S230 (containing only PY83) after aging, consistent with MeV SIMS results showing potential PY13 loss (Figure 3A). Similarly, red inks S324(2) and S324(3) showed aging-related shifts, with S324(3) moving toward the brown ink RACPB cluster (containing PR123, structurally similar to PO43 in S324(3)). This suggests PR112 fading and increased PO43 prominence. In violet inks containing PB15:1 with PR122 (S410), or PV23 (S416), data points clustered closer to WNPC ink after aging, potentially highlighting PB15:1’s greater light stability compared to the other SOPs.^59^

#### 3.1.2 Binders and Additives

Py-GC/MS enabled the clear identification of the acrylate monomers in binders and various additives across all four analyzed unaged acrylic ink mock-ups (green S510, RACBG, and RACPG, and blue WNPC) on Japanese paper. Corresponding pyrograms, results, and compound assignments are detailed in SI (Figure S13 and Table S4). MeV SIMS detected additional compounds that were not identified by Py-GC/MS due to their thermal decomposition at 600 °C pyrolysis temperature.

Green inks S510, RACBG, and blue WNPC appeared to contain a terpolymer binder p­(EA/MMA/MA), differing from the p­(EA/MMA) often used in acrylic emulsion paints. The inclusion of MA likely reduced the viscosity and enhanced ink flow. In contrast, in the green ink RACBG, a p­(MA/MMA) copolymer was identified. Furthermore, co-solvents (glutarate esters), humectants (glycol-based), surfactants (polyethoxylated), and plasticizers (bis­(2-ethylhexyl) phthalate) were determined in all four inks. No antioxidants or UV absorbers were detected. However, Elvacite 2046 (a 1:1 isobutyl methacrylate/*n*-butyl methacrylate in methyl isobutyl ketone) was found in the blue WNPC ink, a binder known to increase gloss and improve UV resistance.^70^ This suggests that WNPC may be among the most stable inks investigated.

MeV SIMS additionally identified poly­(ethylene glycol) (PEG) in Rotring A.C.P. inks (green RACPG, and brown RACPB), which was not observed by Py-GC/MS due to its decomposing into low-molecular-weight compounds such as formaldehyde and ethanol, even at temperatures as low as 350 °C.^71^ MeV SIMS showed nine evenly spaced peaks (*m/z* 780–1140, Δ44 Da), corresponding to repetitive units of −CH_2_CH_2_O– in PEG, consistent with the fragmentation pattern reported by Shen et al. using TOF-SIMS.^72^

### 3.2 Acrylic Ink Mock-Ups on Watercolor Paper

One year of exposure under varied museum lighting affected acrylic ink mock-ups on watercolor paper (yellow RACGY and red S324(3) inks), correlating well with previously MFT-aged mock-ups. VIS reflectance spectroscopy gave an initial overview of changes, while PCA–LDA and MeV SIMS emphasized colorant-level differences, with MeV SIMS also detecting surface chemical changes. Overall, both inks showed the greatest stability on the museum wall, behind 82% UV-protective glass.

VIS reflectance spectra of RACGY mock-ups (Figure 7A) initially showed the PY83/PY13 mixture. Mock-ups in the shop window (direct sunlight) and the INTK drawer (dark, unaged reference) exhibited a ∼10 nm hypsochromic shift matching PY83’s spectral curve,^60^ confirming PY83’s superior stability over PY13. Changes in S324(3) mock-ups were more pronounced (Figure 7B): the museum wall-displayed mock-up showed the least alteration, as expected due to 82% UV glass protection and a windowless room, whereas the shop-window mock-up was most affected. In the latter, reflectance increased by 10%, and a hypsochromic shift (inflection point from ∼560 to ∼550 nm) indicated greater contribution from the more stable PO43 over PR112.^60^ Thus, PR112 underwent significant fading, consistent with the findings of Guarnieri et al.^73^ Δ*E*_00_ between wall- and window-aged mock-ups was 3.69 for the yellow ink (moderate fading) and 10.18 for the red ink (substantial fading).

**Figure 7:**
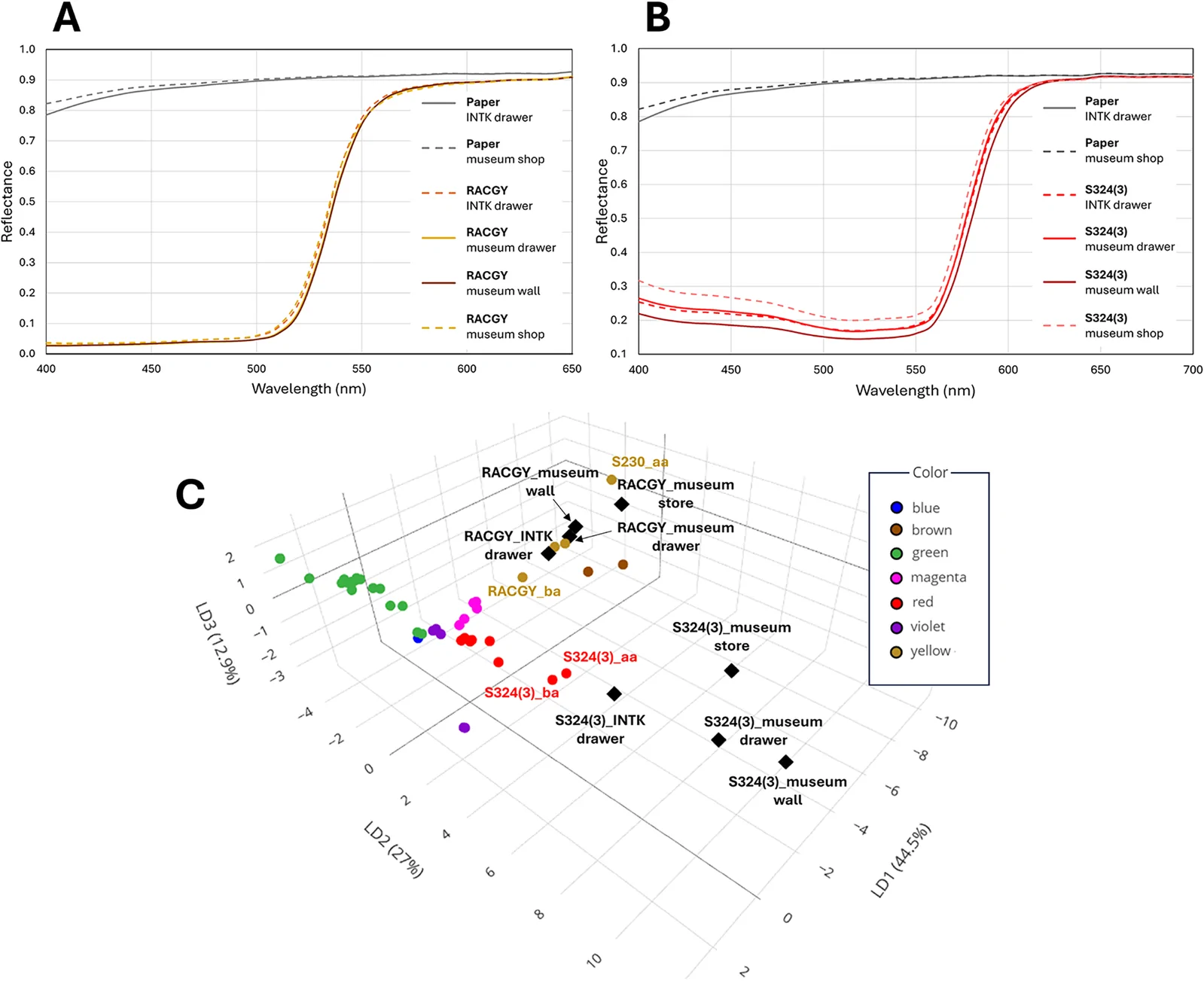
VIS reflectance spectra of the acrylic ink mock-ups on watercolor paper of the yellow RACGY (*Goldgelb*, Rotring ArtiColor) and red S324(3) (*Zinnoberrot*, Schmincke AEROCOLOR) inks (shown in A and B, respectively), and the result of introducing their Raman spectra (785 nm) into the 785 nm laser PCA–LDA model created for the acrylic ink mock-ups on Japanese paper (C). In panel (C), the acrylic ink mock-ups on watercolor paper are highlighted through black rhomboids. In the data point labels of the original models, *ba* denotes *before aging* and *aa* denotes *after aging*.

The 785 nm PCA–LDA model (Figure 7C) correctly classified all four RACGY mock-ups and three of four S324(3) mock-ups. Notably, the shop-window RACGY mock-up clustered closely with the MFT-aged S230 yellow ink (PY83 only), while the museum-wall and drawer RACGY mock-ups clustered near the (unaged) INTK drawer mock-up. This suggests that current display guidelines are effective at preserving such acrylic inks. The shop-window S324(3) mock-up was classified as brown, likely shifting toward the RACPB (PR123) cluster due to PO43’s structural similarity to PR123, consistent with PO43 degrading more slowly than PR112. However, no comparable trend was observed for the S324(3) museum-wall and drawer mock-ups, possibly reflecting subtle differences in Raman response: PR112 (naphthol AS) exhibits fluorescence under Raman analysis, leading to lower-quality spectra, unlike the PY83 and PY13 (diarylides) in RACGY, which present consistent spectral background and peak intensities, allowing for proper comparison.

MeV SIMS supported the spectrophotometric results. In RACGY, PY13 was absent in the museum-displayed mock-ups, agreeing with the PCA–LDA models indicating lower stability of PY13 versus PY83 (Figure 7C). This finding aligns with Herbst et al.,^59^ who report PY13 ranks at level 3–4 on the Blue Wool Scale, whereas more stable PY83 ranks at level 5. After display, PY83 was primarily identified via its fragment ion *m/z* 187/189; only the museum-wall mock-up retained its [M]^+^ ion (*m/z* 816/818) and the diarylide marker ion (*m/z* 252/254), suggesting decreased surface pigment concentration, likely from degradation or overlayer effects (e.g., surfactant migration). For S324(3), relative PR112 and PO43 [M]^+^ ion intensities remained largely unchanged across display locations. The PO43 increase suggested by VIS reflectance data and the PCA–LDA model was not observed by MeV SIMS, possibly reflecting surface heterogeneity, pigment agglomeration, or surface migration, creating small, localized areas difficult to analyze. Regarding surface migration, museum-displayed mock-ups showed a peak at *m/z* 506 (Figure S12, SI), attributed to the [M + H – CO]^+^ ion of hydrolyzed alkyl ketene dimer (AKD), a common paper sizing agent that renders cellulose fibers hydrophobic.^74,75^ The watercolor paper substrate itself showed the protonated AKD molecule [C_36_H_69_O_2_]^+^ at *m/z* 534. Since AKD hydrolyzes and migrates to the surface under ambient conditions (23 °C, 50% RH),^75^ it might partially cover SOP particles after museum display, which reduces SOP secondary ion yield and overall detectability.

### 3.3 Application of the Analytical Protocol to a Real Sample

Since μ-Raman with the PCA–LDA model (especially the 785 nm laser dataset) and MeV SIMS proved robust for identifying SOPs and characterizing acrylic ink photoaging, the real blue ink-on-paper sample (S2B1) from Erich Sokol’s caricature (Figure 8A) was analyzed. In the 785 nm laser PCA-LDA model, the ink of S2B1 was correctly classified as blue and clustered near WNPC (blue ink) containing PB15:3. This indicated that S2B1 also contains the same PB15 polymorph (Figure 8B). In the MeV SIMS spectrum, a peak at *m/z* 575 corresponding to the [M]^+^ ion of a PB15 polymorph was observed (Figure 8C). However, the [M + Cl]^+^ ion typical of PB15:1 was not identified, confirming the presence of PB15:3. A peak at *m/z* 191/193, corresponding to a [C_8_H_4_CuN_2_]^+^ fragment ion, was detected and may further support the presence of PB15:3, known for its high light stability. Overall, this result demonstrates the suitability of the analytical protocol for real, minute samples from cultural heritage objects containing pigment-based acrylic inks.

**Figure 8:**
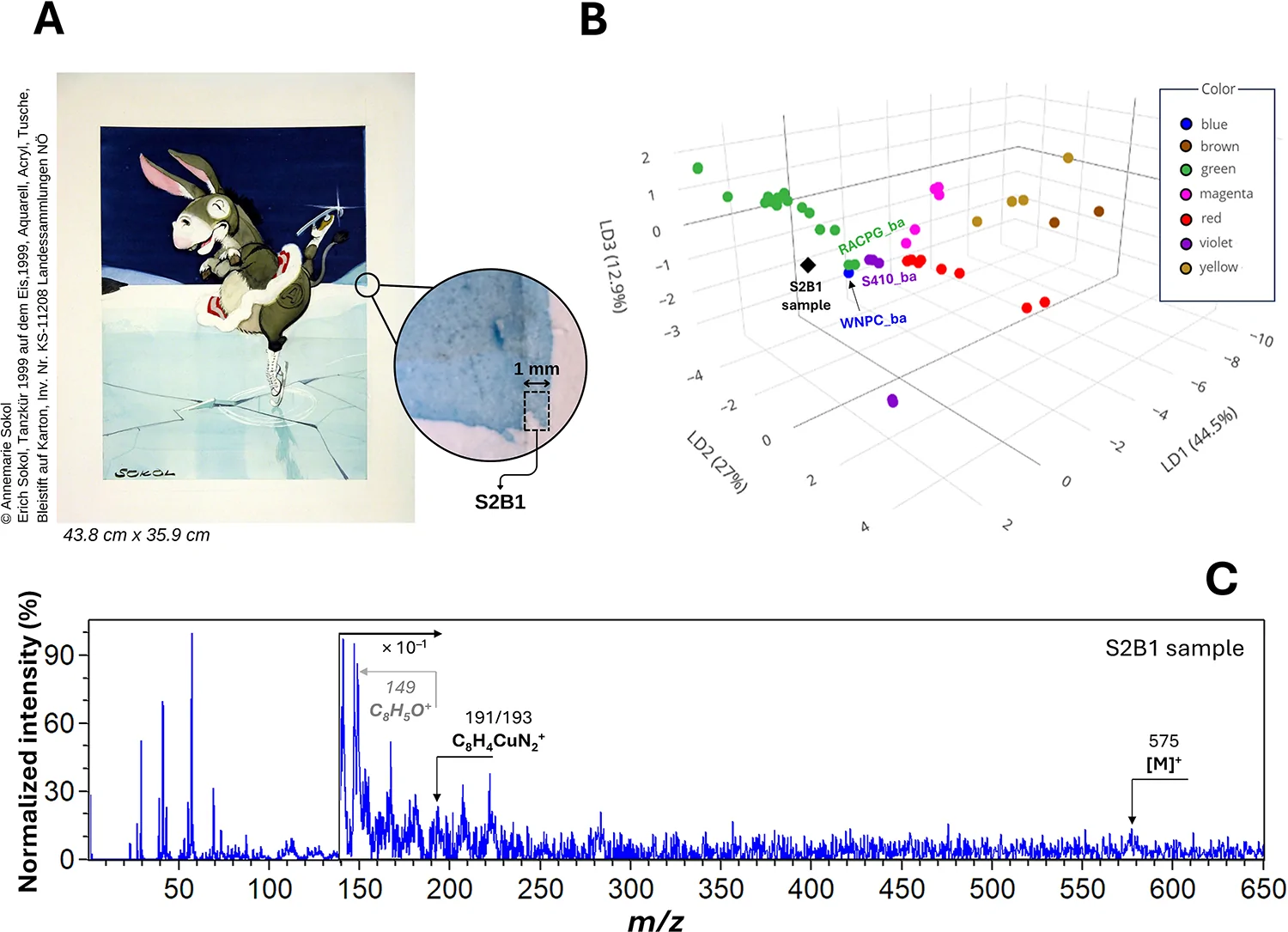
Real sample of a blue ink (S2B1) from an original caricature (Erich Sokol, *Tanzkür 1999 auf dem Eis*,1999, watercolor, acrylic, ink, pencil on cardboard, inv. no. KS-11208, Landessammlungen NÖ © Annemarie Sokol) (A), analyzed using the developed analytical protocol for identifying SOPs in acrylic inks. Panel (B) shows the position of the Raman spectrum of the sample (as a black rhomboid) within the PCA–LDA model (785 nm); in the data point labels, *ba* denotes *before aging*. Panel (C) displays the 8 MeV Si^4+^ MeV SIMS spectrum. Both panels (B) and (C) identify the SOP in S2B1 as a polymorph of PB15 (phthalocyanine blue).

## 4 Conclusions

This study presents the first systematic investigation of acrylic inks in a heritage science context. Twenty-seven acrylic inks from the original bottles used by Erich Sokol (1933–2003), produced by Schmincke, Rotring and Winsor & Newton, were analyzed to develop a minimally invasive analytical protocol for identifying synthetic organic pigments (SOPs) and assessing their photodegradation in acrylic inks on paper. The inks on paper were photoaged through microfading and displayed during a one-year temporary exhibition, *I LOVE DEIX*, at the Karikaturmuseum Krems, Austria (February 2024–February 2025).

The analytical approach combined μ-Raman spectroscopy and MeV SIMS, enabling direct analysis of ink on paper samples without chemical pretreatment or sample consumption, an essential advantage for cultural heritage objects, where samples are minute and highly valuable. PCA–LDA models applied to Raman datasets proved effective for rapid ink classification, detection of pigment mixtures, and evaluation of aging-related changes. Py-GC/MS provided additional insight into binders and additives potentially influencing photodegradation. Investigated inks contain moderately to highly lightfast SOPs, including phthalocyanine, naphthol AS, diarylide, quinacridone, monoazo, dioxazine, perylene, and perinone pigments, often present as mixtures. Notable formulation differences were detected, such as the replacement of PO5 by the more lightfast PO43 in *Zinnoberrot* inks from Schmincke AEROCOLOR, demonstrating the discriminatory power of the analytical protocol. The presence of Elvacite 2046 as an additional binder suggests enhanced UV resistance in the Winsor & Newton ink.

MFT aging using LED light (2733 K, spectral range: 400–750 nm) seemed to have caused no substantial SOP photodegradation in the inks. MeV SIMS revealed a decrease in relative intensities of SOP molecular ions and characteristic fragments; however, further systematic studies are needed to clarify whether this outcome derives only from SOP degradation or also from other factors like pigment agglomeration or surface migration causing overlayer effects. In some pigment mixtures, this yield decrease manifested as one SOP evading detection, such as PY13 in the yellow ink RACGY. Finally, the protocol’s efficiency was successfully tested on a real sample from Erich Sokol’s “*Tanzkür 1999 auf dem Eis*” (1999), where blue ink was found to contain a phthalocyanine PB15:x polymorph (likely PB15:3).

Overall, the developed analytical protocol demonstrates that caricature display on the wall behind 82%-UV-protective glass, or in a drawer with only temporary light exposure, effectively preserves acrylic ink color. This confirms the protocol’s value as a practical tool for guiding future conservation decisions on display and storage. The CIE157:2004 lighting standard classifies works on paper as highly light-sensitive, restricting illuminance to 50 lux (0.12 Mlux·h/year).^39,76^ However, since SOPs identified here (e.g., PB15:x, PG7, PG36, PV23, PR123) are moderately to highly lightfast, and no significant aging effects were observed on tested mock-ups, acrylic inks could be classified as moderately light-sensitive in this case. Despite these positive outcomes, decisions on whether and how long to display real works on paper with acrylic inks depend not only on constituent materials, but also on the entire condition of the objects themselves; poor condition would warrant stricter conservation guidelines.

## Supplementary Material



## Notes

Original ink bottles and corresponding mock-ups, additional MeV SIMS spectra, and detailed Py-GC/MS results (PDF)
