# Supplementary material for: Assessing Acrylic Inks: A Study of Colorants and Lightfastness
Source: ACS Omega. 2026 Sep 22;11(39):58941–57. doi: 10.1021/acsomega.6c04091 (PMC13648065; doi:10.1021/acsomega.6c04091)
Supplement: Supplementary file 1 [file ao6c04091_si_001.pdf]

SUPPORTING INFORMATION

Assessing Acrylic Inks: A Study of Colorants and Lightfastness

Teodora Raicu[1,*], Matea Krmpotić[2], Iva Bogdanović Radović[2], Zdravko Siketić[2],Katja Sterflinger[1], Dubravka Jembrih-Simbürger[1]

[1]Institute of Natural Sciences and Technology in the Arts (INTK), Academy of Fine Arts Vienna, Kelsenstrasse 2, Vienna, A-1030, Austria

[2]Division of Experimental Physics, Laboratory for Ion Beam Interactions, Ruđer Bošković Institute, Bijenička cesta 54, Zagreb, HR-10000, Croatia

*Corresponding author: t.raicu@akbild.ac.at

Table of Contents

Original Ink Bottles and Corresponding Mock-ups 2
Figure S-1. 2
Figure S-2. 3
MeV SIMS Spectra 4
Table S-1 4
Figure S-3. 4
Figure S-4. 5
Figure S-5. 5
Figure S-6. 6
Figure S-7. 6
Figure S-8. 7
Figure S-9. 7
Figure S-10 8
Figure S-11 8
Figure S-12 9
Table S-2 9
Py-GC/MS Analysis and Results 10
Table S-3 10
Figure S-13 12
Table S-4 13
References 14

Original Ink Bottles and Corresponding Mock-ups

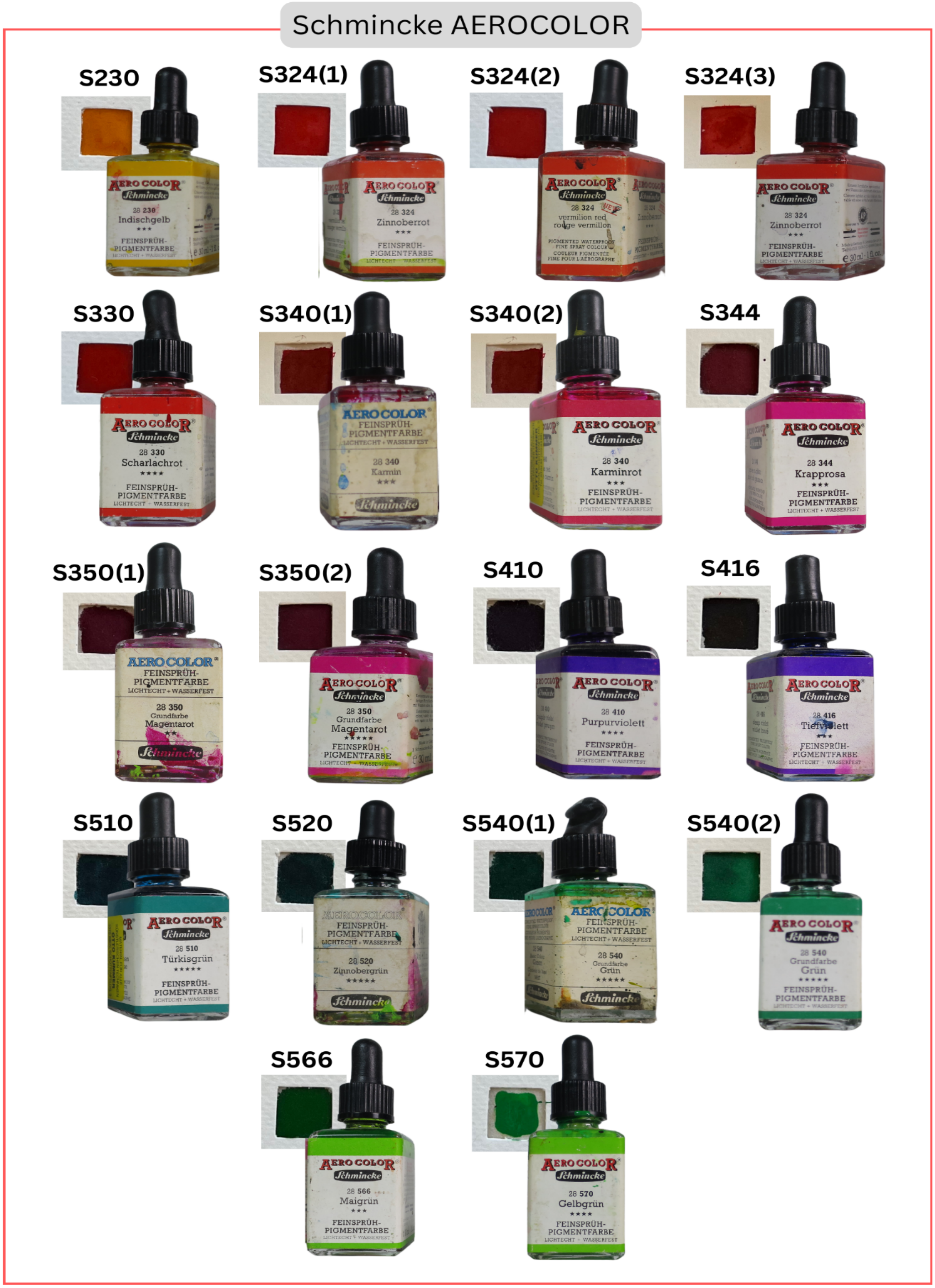

Figure S-1. The Schmincke AEROCOLOR acrylic ink bottles and corresponding ink mock-ups on Japanese paper along with assigned ink codes (shown in each upper-left corner).

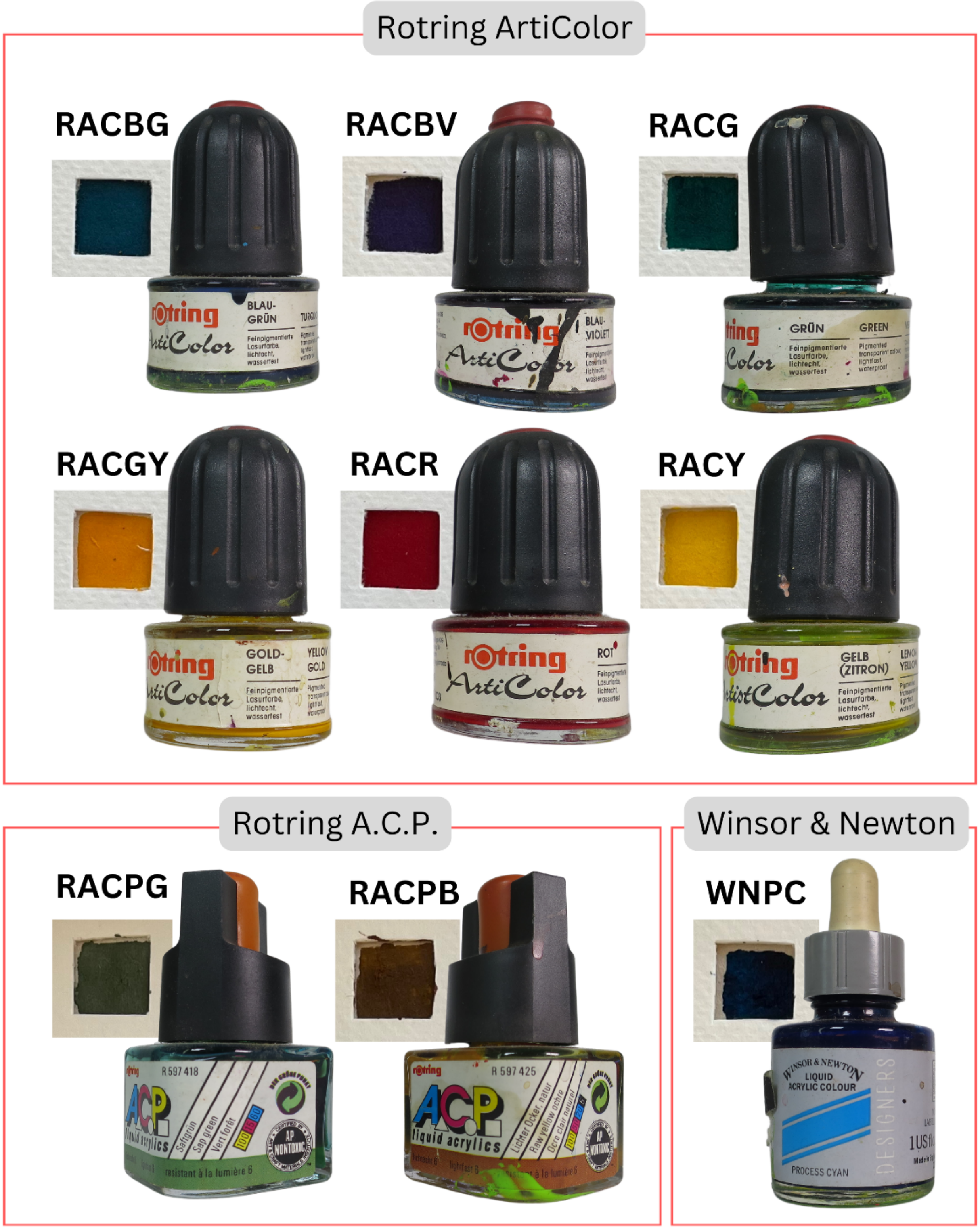

Figure S-2. The Rotring (ArtiColor and A.C.P.) and Winsor & Newton (W&N) acrylic ink bottles and corresponding ink mock-ups on Japanese paper along with assigned ink codes (shown in each upper-left corner).

MeV SIMS Spectra

Table S-1. Synthetic organic pigment reference powders analyzed by MeV SIMS in positive-ion mode using 8 MeV Si^{4+} primary ions. All pigments originate from INTK's Materials Collection, except for PO43 and PR146, which were acquired from the Hangzhou Mida Chemical Co. (China) and were measured with 5 MeV Si^{4+} beam due to technical restrictions.

Color index name	Color	Chemical class	INTK inv. no. = i.n.	Bottle name	Manufacturer/ Reseller	Chemical formula	Monoisotopic mass (Da)	Molecular weight (g/mol)
**PY12**	yellow	diarylide yellow	172	Heliogelb GWNPh	Bayer, Leverkusen	$C_{32}H_{26}Cl_2N_6O_4$	628.1393	629.5
**PY13**	yellow	diarylide yellow	955	Vynamon Yellow GRES FW	ICI	$C_{36}H_{34}Cl_2N_6O_4$	684.2019	685.6
**PY14**	yellow	diarylide yellow	944	Benzidine Yellow Toner 22-C	Sun Chemical Corporation	$C_{34}H_{30}Cl_2N_6O_4$	656.1706	657.5
**PY83**	yellow	diarylide yellow	1299	Permanentgelb HR extra Pulver (86 0 207)	Farbwerke Hoechst AG	$C_{36}H_{32}Cl_4N_6O_8$	816.1036	818.5
**PO43**	orange	perinone	–	–	Hangzhou Mida Chemical Co.	$C_{26}H_{12}N_4O_2$	412.0960	412.4
**PR7**	red	naphthol AS	565	Monolite Red 4 RHS powder	ICI	$C_{25}H_{19}Cl_2N_3O_2$	463.0854	464.3
**PR146**	red	naphthol AS	–	–	Hangzhou Mida Chemical Co.	$C_{33}H_{27}ClN_4O_6$	610.1619	611.0
**PV23**	violet	dioxazine	1297	Permanentviolett RL extra Pulver (86 4 053)	Farbwerke Hoechst AG	$C_{34}H_{22}Cl_2N_4O_2$	588.1120	589.5

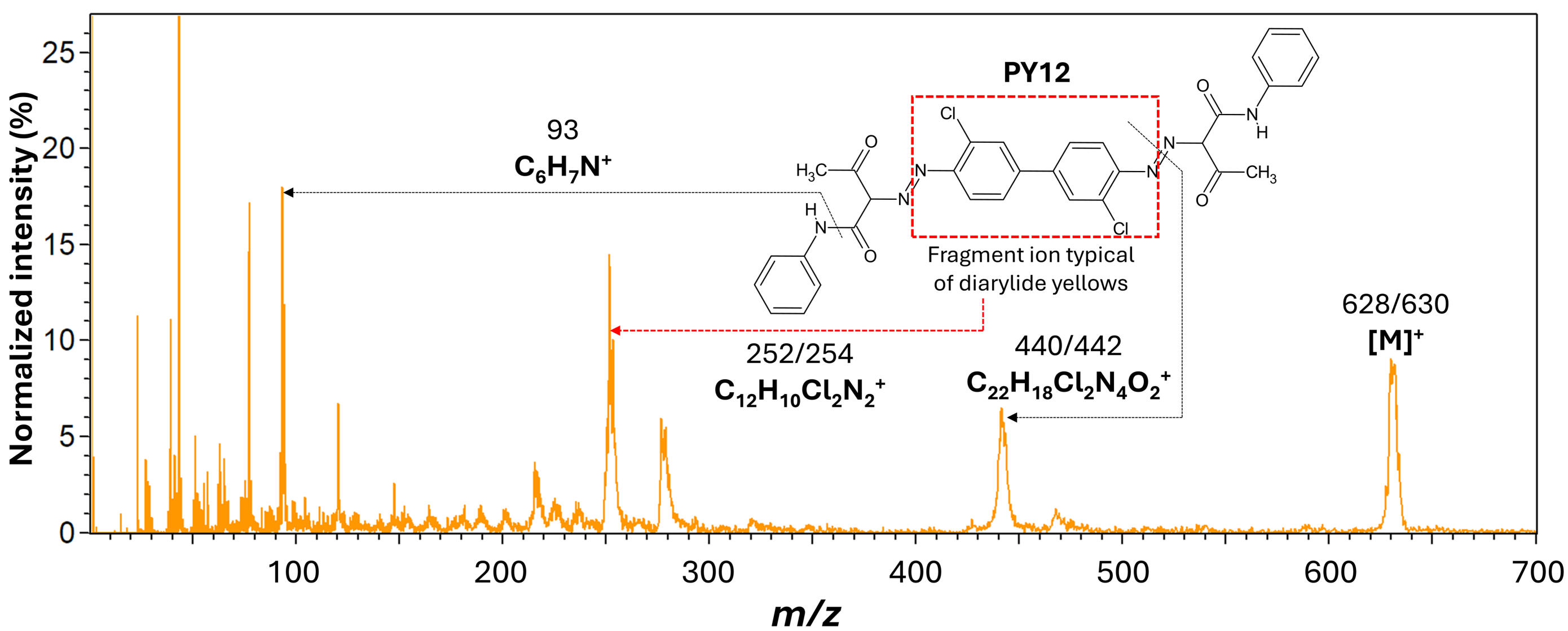

Figure S-3. The positive-ion mode MeV SIMS spectrum (8 MeV Si^{4+}) of PY12 (yellow pigment powder, Heliogelb GWNPh, Bayer, Leverkusen).

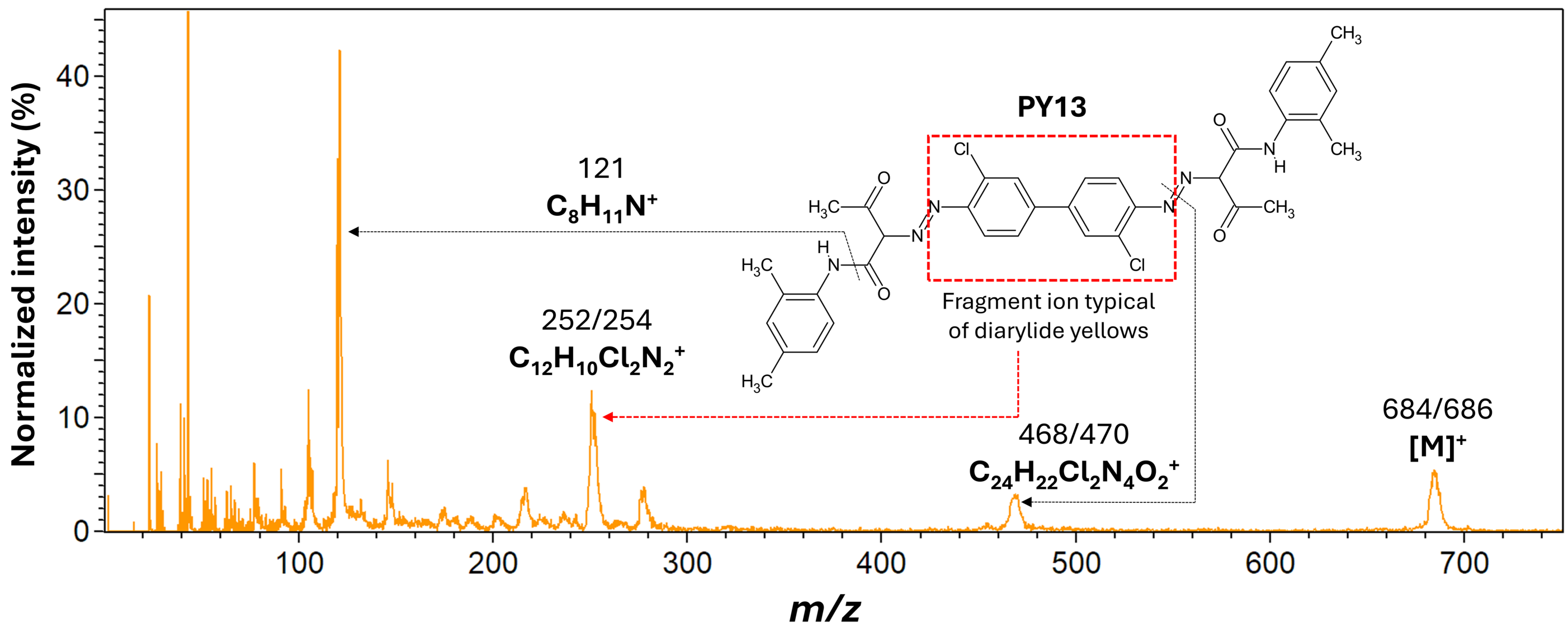

Figure S-4. The positive-ion mode MeV SIMS spectrum (8 MeV Si^{4+}) of PY13 (yellow pigment powder, Vynamon Yellow GRES FW, ICI).

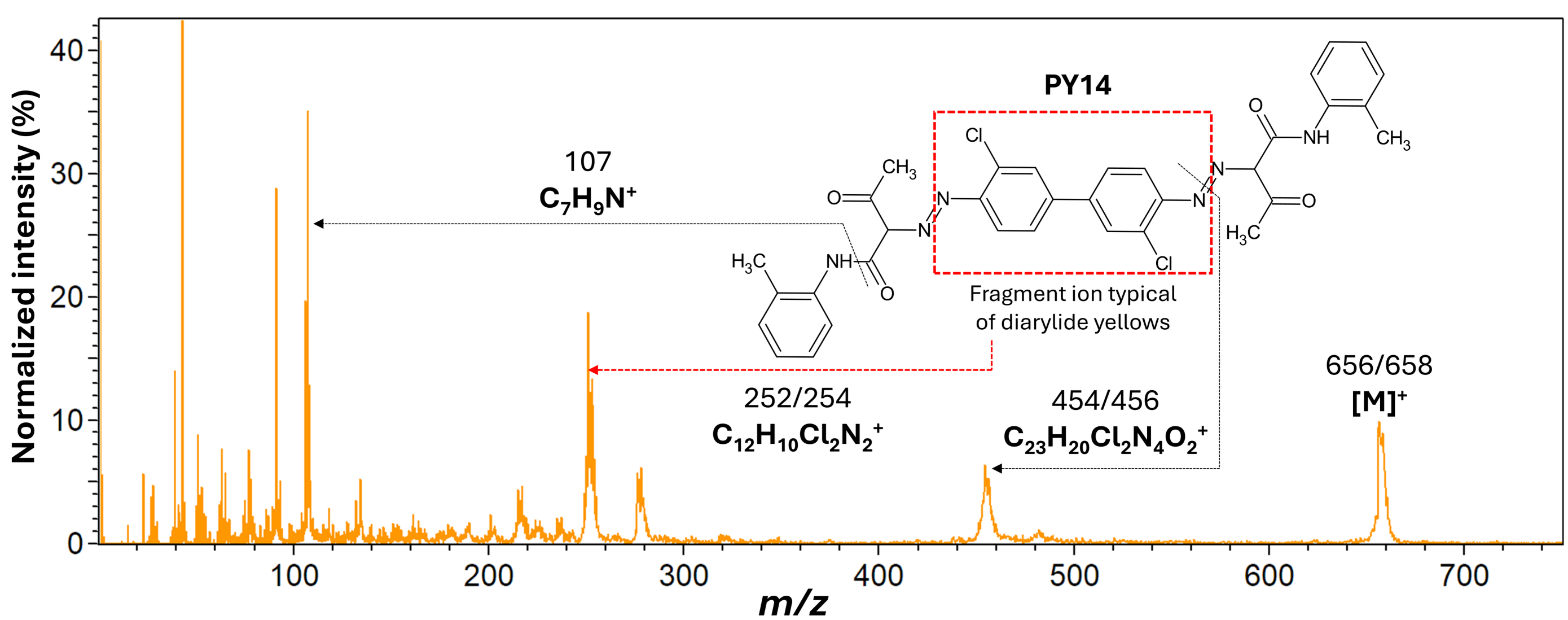

Figure S-5. The positive-ion mode MeV SIMS spectrum (8 MeV Si^{4+}) of PY14 (yellow pigment powder, Benzidine Yellow Toner 22-C, Sun Chemical Corporation).

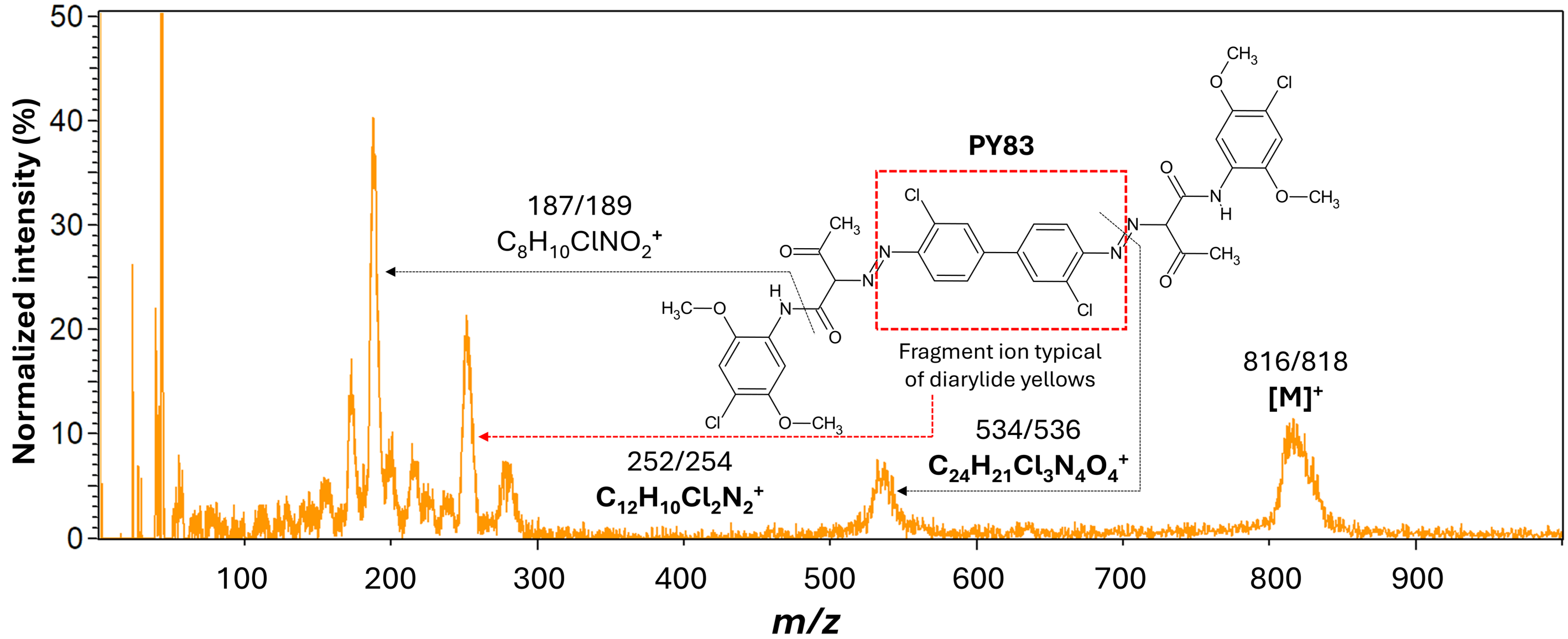

Figure S-6. The positive-ion mode MeV SIMS spectrum (8 MeV Si^{4+}) of PY83 (yellow pigment powder, Permanentgelb HR extra Pulver (86 0 207), Farbwerke Hoechst AG).

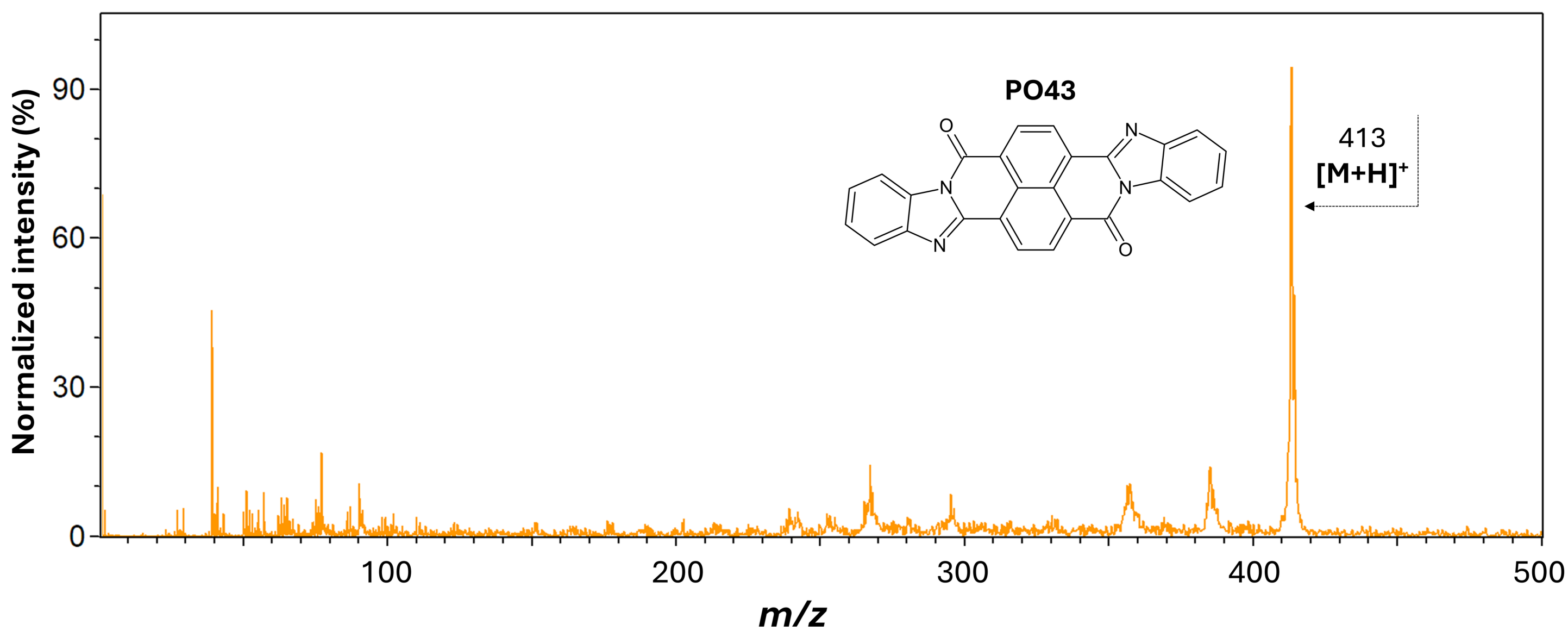

Figure S-7. The positive-ion mode MeV SIMS spectrum (5 MeV Si^{4+}) of PO43 (orange pigment powder, Hangzhou Mida Chemical Co.).

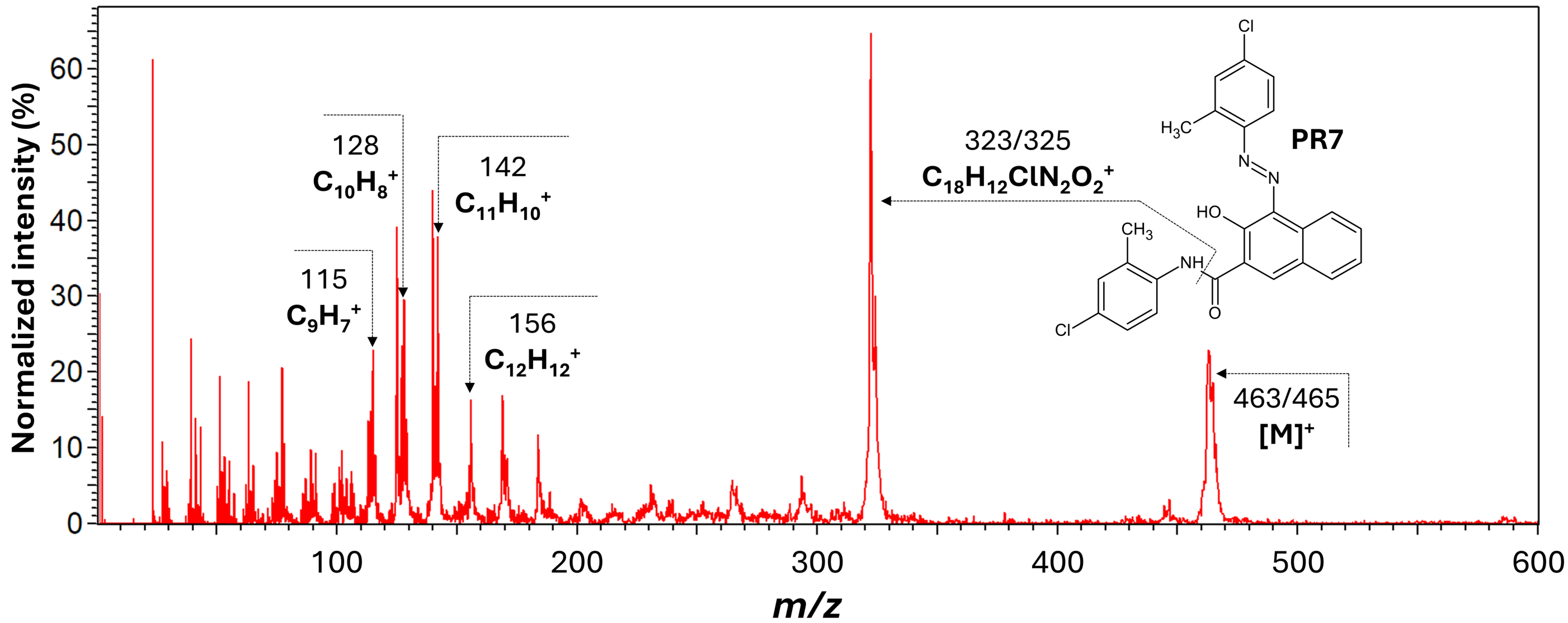

Figure S-8. The positive-ion mode MeV SIMS spectrum (8 MeV Si^{4+}) of PR7 (red pigment powder, Monolite Red 4 RHS powder, ICI).

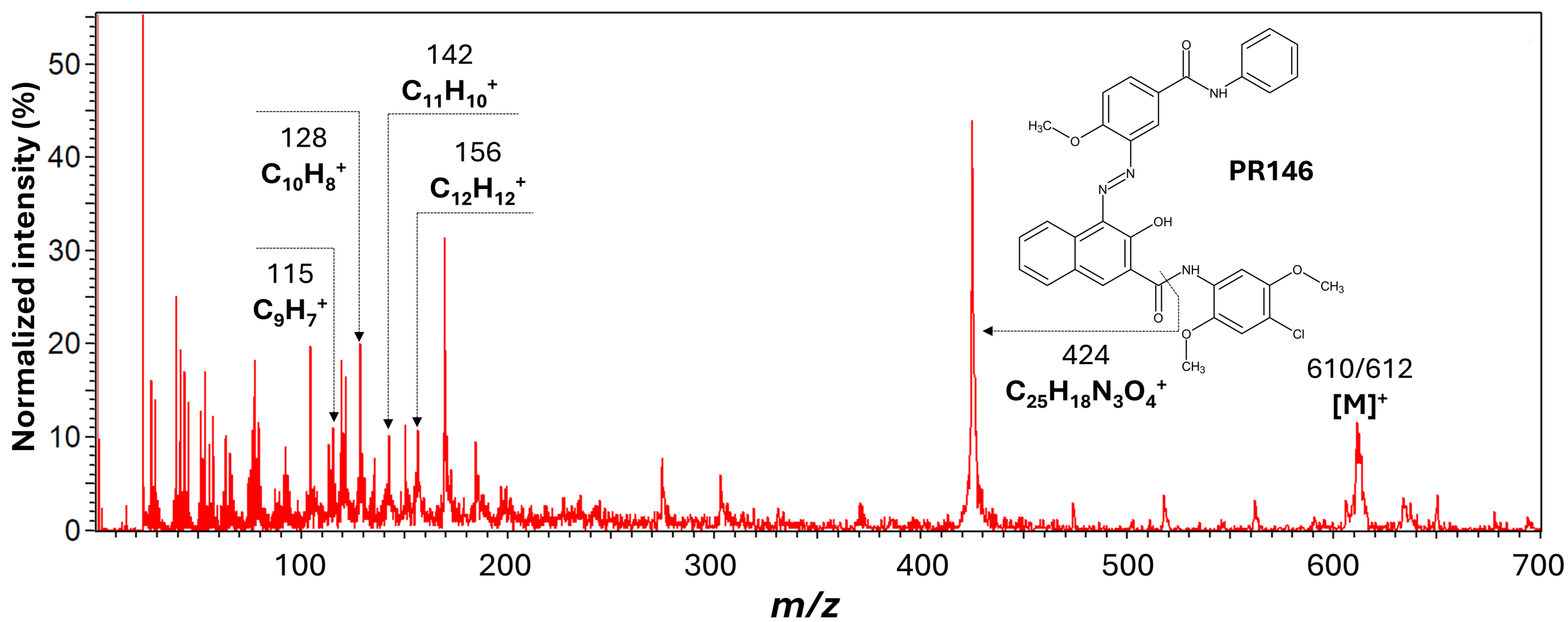

Figure S-9. The positive-ion mode MeV SIMS spectrum (5 MeV Si^{4+}) of PR146 (red pigment powder, Hangzhou Mida Chemical Co.).

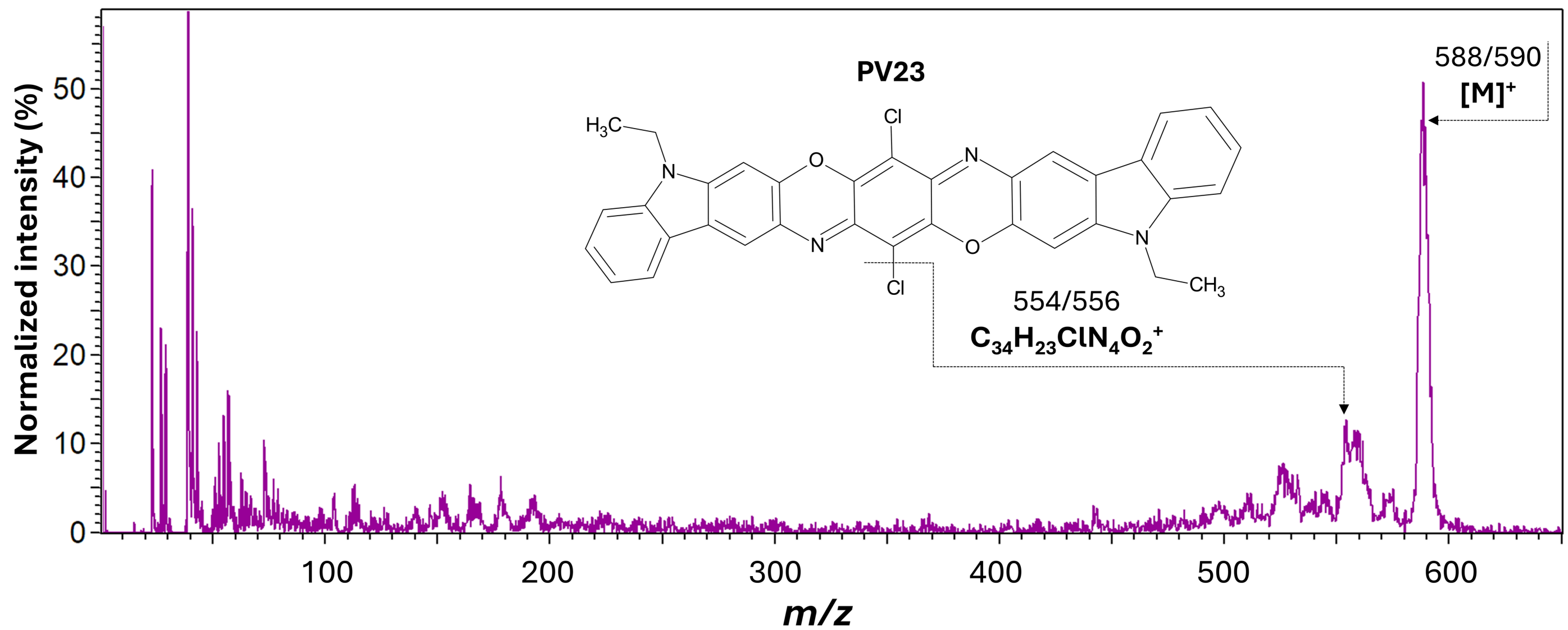

Figure S-10. The positive-ion mode MeV SIMS spectrum (8 MeV Si^{4+}) of PV23 (violet pigment powder, Permanentviolett RL extra Pulver (86 4 053), Farbwerke Hoechst AG).

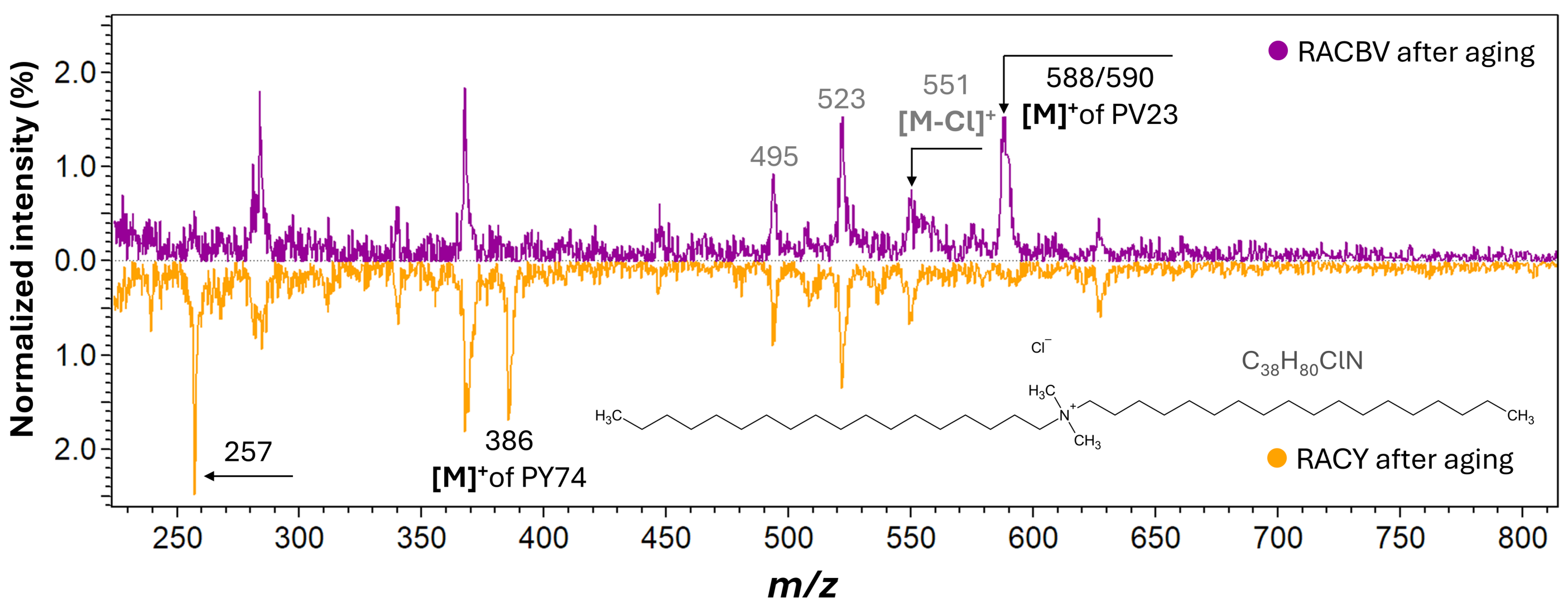

Figure S-11. Comparison of positive-ion mode MeV SIMS spectra (8 MeV Si^{4+}) of the blue-violet acrylic ink RACBV (*Blau-violett*, Rotring ArtiColor) and yellow ink RACY (*Gelb (zitron)*, Rotring ArtiColor) on Japanese paper after MFT aging in the relevant *m/z* region 230–810. The appearance of new peaks at *m/z* 495, 523, and 551 in both inks is attributed to dimethyldioctadecylammonium chloride ($C_{38}H_{80}ClN$), a cationic surfactant. Peaks at *m/z* 257 and 284 were also observed after aging, but they could not be assigned to a specific compound so far.

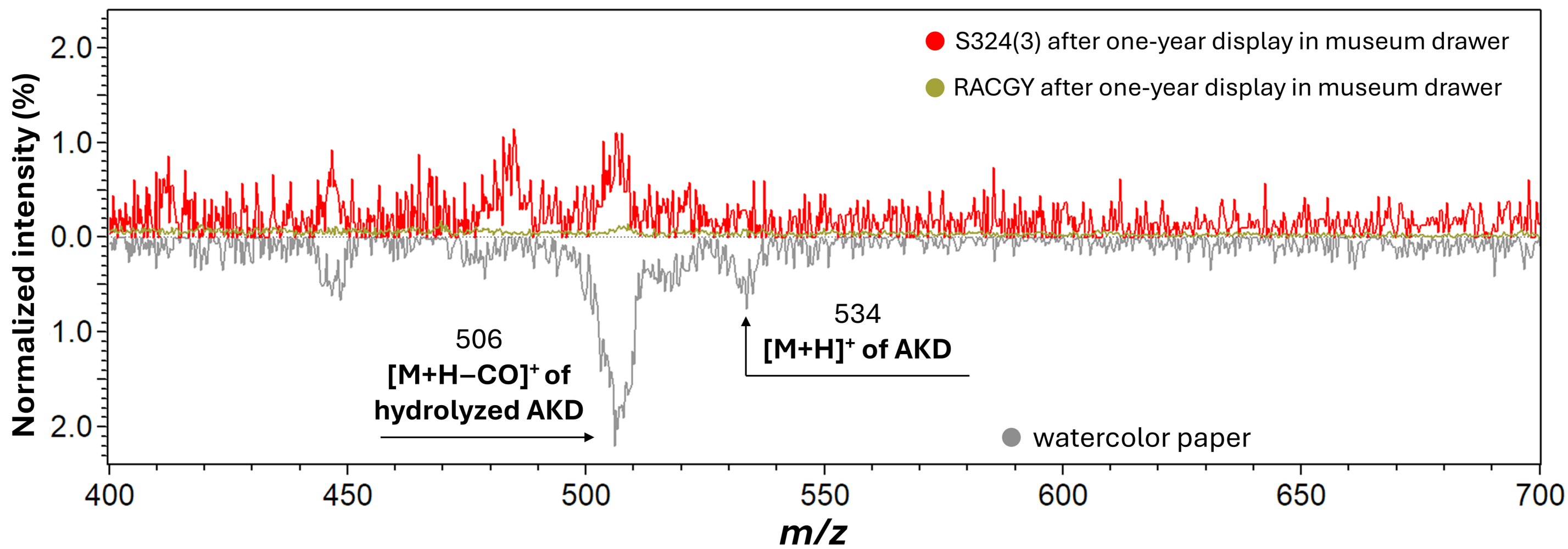

Figure S-12. Comparison of the positive-ion mode MeV SIMS spectra (8 MeV Si^{4+}) of red S324(3) (cinnabar red, *Zinnoberrot*, Schmincke AEROCOLOR) and yellow acrylic ink RACGY (golden yellow, *Gold-gelb*, Rotring ArtiColor) on watercolor paper after one-year exposition in the museum drawer (*m/z* region 400–700). After exposition peaks at *m/z* 506 and 534 are detected and attributed to alkyl ketene dimer (AKD), a sizing agent originating from the watercolor paper.

Table S-2. Characteristic ions of SOPs identified in reference pigment powders by MeV SIMS (8 MeV Si^{4+}).

Color Index Name	Chemical class	*m/z*	FWHM (full-width half-maximum)	Molecular ion/ Pronated molecule/ Fragment ion	Fragment ion origin
**PO43***	perinone	413	0.8	$[M+H]^+$	--
**PR7**	naphthol AS	115	0.4	$C_9H_7^+$	–
		128	0.5	$C_{10}H_8^+$	–
		142	0.5	$C_{11}H_{10}^+$	–
		156	0.5	$C_{12}H_{12}^+$	–
		323/325	1.2	$C_{18}H_{12}ClN_2O_2^+$	amide bond cleavage
		463/465	3.0	$[M]^+$	–
**PR146***	naphthol AS	115	0.4	$C_9H_7^+$	–
		128	0.3	$C_{10}H_8^+$	–
		142	0.5	$C_{11}H_{10}^+$	–
		156	0.6	$C_{12}H_{12}^+$	–
		424	1.1	$C_{25}H_{18}N_3O_4^+$	amide bond cleavage
		610/612	3.0	$[M]^+$	–
**PV23**	dioxazine	554/556	9.7	$C_{34}H_{23}ClN_4O_2^+$	$[M–Cl]^+$
		588/590	4.7	$[M]^+$	–
**PY12**	diarylide yellow	93	0.4	$C_6H_7N^+$	amide bond cleavage

Color Index Name	Chemical class	*m/z*	FWHM (full-width half-maximum)	Molecular ion/ Pronated molecule/ Fragment ion	Fragment ion origin
		252/254	0.9	$C_{12}H_{10}Cl_2N_2^+$	double azo bond cleavage
		440/442	4.6	$C_{22}H_{18}Cl_2N_4O_2^+$	azo bond cleavage
		628/630	4.4	$[M]^+$	–
**PY13**	diarylide yellow	121	0.7	$C_8H_{11}N^+$	amide bond cleavage
		252/254	4.1	$C_{12}H_{10}Cl_2N_2^+$	double azo bond cleavage
		468/470	5.3	$C_{24}H_{22}Cl_2N_4O_2^+$	azo bond cleavage
		684/686	5.8	$[M]^+$	–
**PY14**	diarylide yellow	107	0.3	$C_7H_9N^+$	amide bond cleavage
		252/254	0.6	$C_{12}H_{10}Cl_2N_2^+$	double azo bond cleavage
		454/456	4.0	$C_{23}H_{20}Cl_2N_4O_2^+$	azo bond cleavage
		656/658	4.0	$[M]^+$	–
**PY83**	diarylide yellow	187/189	6.1	$C_8H_{10}ClNO_2^+$	amide bond cleavage
		252/254	6.3	$C_{12}H_{10}Cl_2N_2^+$	double azo bond cleavage
		534/536	10.2	$C_{24}H_{21}Cl_3N_4O_4^+$	azo bond cleavage
		816/818	16.2	$[M]^+$	–

*measured with 5 MeV Si^{4+} ion beam

Py-GC/MS Analysis and Results

Table S-3. Single-shot (SS) Py-GC/MS parameters

Instrument unit	Parameters	Settings
Pyrolyzer	Furnace temperature	600 °C
	Interface temperature	280 °C
GC	Injection temperature	250 °C
	Column oven temperature	40 °C (5 min hold) – 280 °C (6 °C /min, 3 min hold)
	Injection mode	Split (split ratio 1:50)
	Carrier gas	He (5.0 grade, 99.999%)
	Column flow	0.6 mL/min
	Flow control mode	Linear Velocity
	Pressure	37.8 kPa
	Total flow	33.6 mL/min
MS	Ion source temperature	200 °C

Instrument unit	Parameters	Settings
	Ion source interface temperature	280 °C
	Ionization method	Electron ionization (EI), 70 eV
	Scan range	*m/z* 50–750
	Scan speed	1428 u/sec
	Acquisition mode	Scan

Copolymers of **acrylate monomers** were detected in all four selected inks (green S510, RACBG, and RACPG, and blue WNPC) (Figure S-13A). S510, RACPG, and WNPC inks contained **methyl acrylate** (MA, RT 3.17 min), **ethyl acrylate** (EA, RT 4.42 min), and **methyl methacrylate** (MMA, RT 4.78 min). Sesquimers, dimers and trimers—typical pyrolysis products of these monomers [1,2]—were also observed, with the EA-MMA sesquimer (EA-MMA S, RT 21.0 min), and trimer species (EA-MMA T, RT 33.24 min) detected only in green S510 and blue WNPC inks. Ethyl methacrylate (EMA, RT 7.14 min) appeared as a secondary product of p(EA) pyrolysis rather than a monomer present alongside EA and MMA [3]. The green RACBG ink lacked EA, but contained MA and MMA, suggesting a p(MA/MMA) binder. A strong peak at RT 30.07 min (*m/z* 59, 79, 134, 167, 194, 227), was assigned to the MA trimer (MA T, RT 30.08 min), consistent with the findings of Moldoveanu [4]. Since MA and its trimer were also detected in the S510, RACPG, and WNPC inks, albeit at a lower intensity, the use of a **terpolymer p(EA/MMA/MA)** is plausible. This finding is significant, as such an acrylic medium has not been previously reported for acrylic emulsion paints. The inclusion of two soft monomers (EA, MA) alongside MMA may have been intended to reduce viscosity and improve flow. The absence of n-butyl acrylate fragments assignable to p(nBA/MMA) indicates these inks were manufactured before or around 1980, when p(nBA/MMA) became common [5].

In addition to binders, Py-GC/MS revealed additives, including co-solvents, humectants, surfactants and plasticizers. **Glutarate esters** were detected in all four inks—dimethyl glutarate (DMG, RT 18.22 min) in RACBG, RACPG, and WNPC, and diethyl glutarate (DEG, RT 21.84 min) in S510—consistent with their reported use as **co-solvents** (diluents) for acrylic resins [6]. **Octyl phenol polyethoxy ethanol** (OP, RT 28.95 min) was detected in trace amounts in the green S510 ink, confirming its use as a typical **surfactant** in p(EA/MMA) emulsions [1]. RACPG ink contained **triethylene glycol monododecyl ether** (TEGMO, RT 40.05 min), a glycol-based **humectant** that prevents premature ink drying [7], and **oleyl alcohol methyl ether** (OAME, RT 36.86 min) possibly serving a similar function. Traces of **bis(2-ethylhexyl phthalate)** (DEHP, RT 43.90 min) found in all inks suggest a minor use of **plasticizer**.

Among the four inks, the **blue WNPC** (Winsor & Newton) contained the broadest range of additives. Two nonionic **surfactants** were also identified: **Surfynol 61** (S61, 3,5-dimethyl-1-hexyn-3-ol, RT 9.75 min), a liquid **wetting agent and defoamer**, and **Surfynol 104** (S104, 2,4,7,9-tetramethyl-5-decyne-4,7-diol, RT 24.99 min), a solid, **hydrophobic surfactant** typically dissolved in ethers, alcohols or glycols [8,9]. The additional detection of dipropylene glycol monomethyl ether (DPM, RT 14.39 min) indicates the use of **Surfynol 104 DPM**. This combination enhances pigment wetting, foam control, and flow, while improving water resistance, which may explain its recommended use in inks rather than paints [9,10]. **Methyl isobutyl ketone** (MIK, RT 5.53 min) was identified as a **solvent** typical for acrylic paints and inks [11]. Using ESCAPE software (Figure S-13B), **isobutyl methacrylate** (iBMA) and **n-butyl methacrylate** (nBMA) were also identified. The co-occurrence of MIK with these methacrylate monomers strongly suggests the presence of **Elvacite 2046**, a 1:1 iBMA/nBMA known to be readily soluble in MIK [12]. In this case, Elvacite 2046 was likely employed not as an acrylic binder but as an additive that enhances gloss and is resistant to UV exposure.

Some **pigment**-related pyrolysis products were also observed. Benzene, 1,3-dimethyl- (BDM, RT 10.00 min) and 1,4-benzenedicarbonitrile (BDC, RT 23.00 min) appeared in all inks, except for the green RACPG. The pyrogram of the

green S510 ink additionally showed 3-chloro-1,2-benzenedicarbonitrile (CBDC, RT 25.35 min), 2,4-dichloro-1,3-benzenedicarbonitrile (DBDC, RT 29.07 min), 1,3,7-trichloronaphthalene (TCN, RT 30.8 min), and tetrachloroisophthalonitrile (TCIP, RT 33.62 min). Since all these compounds contain chlorine, they can be primarily attributed to PG7. DBDC and TCN have been previously linked with the presence of chlorinated phthalocyanines [13].

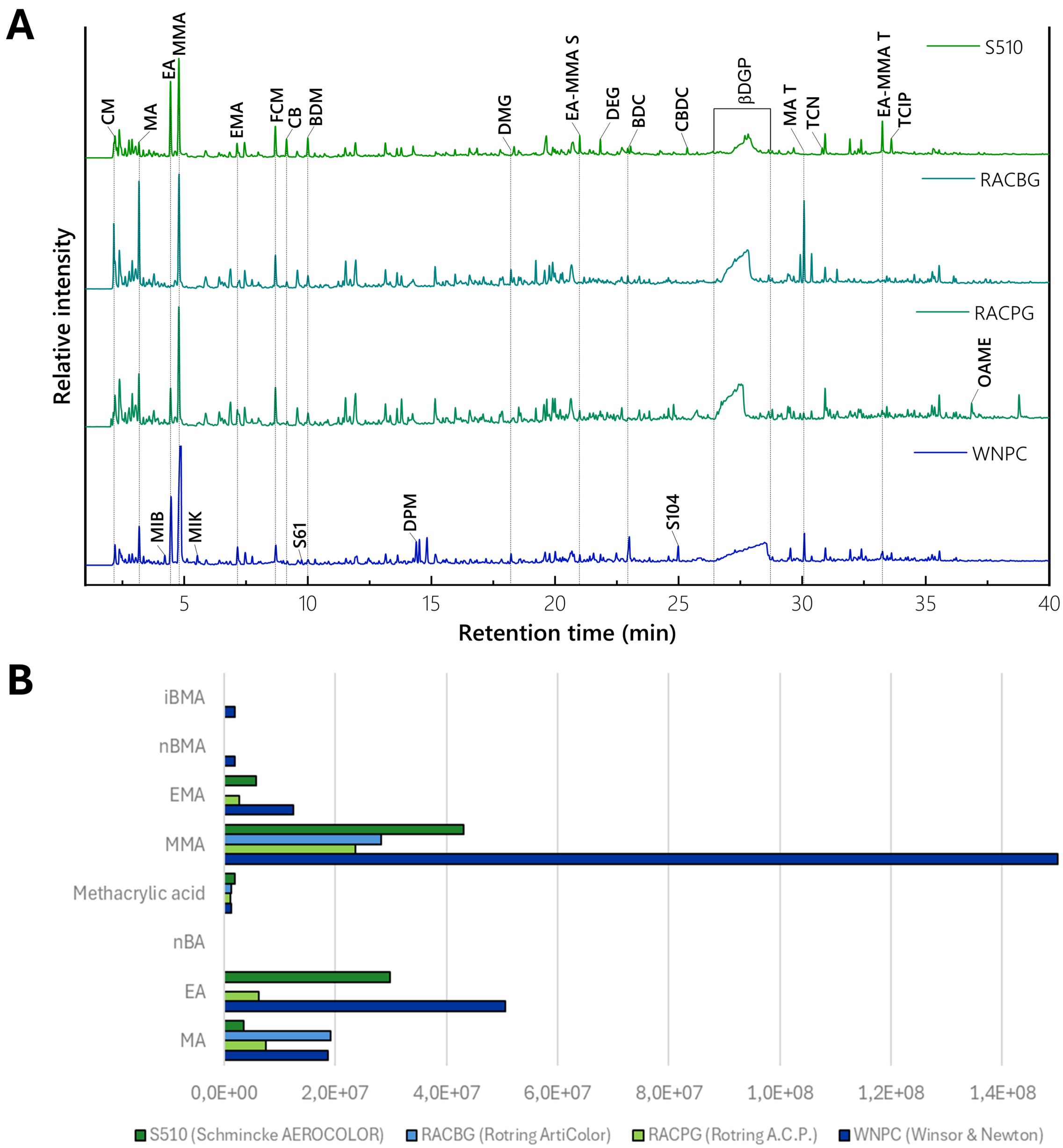

Figure S-13. Identified acrylates and other additives in the four analyzed acrylic inks on Japanese paper: green S510 (Schmincke AEROCOLOR), RACBG (Rotring ArtiColor), and RACPG (Rotring A.C.P.), as well as blue WNPC (W&N). (A) Pyrograms for each ink sample, retention time region shown 2–40 min; the peaks detected are: chloromethane (CM), methyl acrylate (MA), ethyl acrylate (EA), methyl methacrylate (MMA), ethyl methacrylate (EMA), 2-furancarboxaldehyde (FCM), chlorobenzene (CB), benzene, 1,3-dimethyl (BDM), dimethyl glutarate (DMG), EA-MMA sesquimer (EA-MMA S), diethyl glutarate (DEG), 1,4-benzenedicarbonitrile (BDC), 3-chloro-1,2-benzenedicarbonitrile (CBDC), 1,6-anhydro-β-D-glucopyranose (βDGP), 1,3,7-trichloronaphthalene (TCN), EA-MMA trimer (EA-MMA T), and tetrachloroisophthalonitrile (TCIP). RACPG additionally included oleyl alcohol, methyl ether (OAME), and WNPC included methyl isobutyrate (MIB), methyl isobutyl ketone (MIK), Surfynol 61 (S61), dipropylene glycol monomethyl ether (DPM), and Surfynol 104 (S104). (B) Identified acrylate monomers based on the semi-quantitative analysis performed in ESCAPE software [14], highlighting the presence of isobutyl methacrylate (iBMA) and n-butyl methacrylate (nBMA) in WNPC, and the absence of n-butyl acrylate in all four acrylic inks.

Table S-4. Pyrolysis products of green inks S510 (Schmincke AEROCOLOR), RACBG (Rotring ArtiColor), and RACPG (Rotring A.C.P.), as well as of blue WNPC (Winsor & Newton), with their assigned abbreviations. Bold values under the *Main m/z* column indicate the base peaks.

RT (min)	Compound	Green S510 (Schmincke AEROCOLOR)	Green RACBG (Rotring ArtiColor)	Green RACPG (Rotring ACP)	Blue WNPC (Winsor & Newton)	Main *m/z*
**2.15**	Chloromethane (CM)	X	X	–	–	**50**, 64
**3.17**	Methyl acrylate (MA)	X	X	X	X	**55**, 85
**4.28**	Methyl isobutyrate (MIB)	X	X	X	X	59, **71**, 87, 102
**4.42**	Ethyl acrylate (EA)	X	–	X	X	**55**, 99
**4.78**	Methyl methacrylate (MMA)	X	X	X	X	**69**, 100
**5.53**	Methyl isobutyl ketone (MIK)	–	–	–	X	**58**, 85, 100
**7.14**	Ethyl methacrylate (EMA)	X	–	X	X	**69**, 99
**8.7**	2-Furancarboxaldehyde (FCM)	X	X	X	X	67, **96**
**9.14**	Chlorobenzene (CB)	X	X	–	–	77, **112**
**9.75**	3,5-dimethyl-1-hexyn-3-ol (Surfynol 61) (S61)	–	–	–	X	**69**, 111
**10.00**	Benzene, 1,3-dimethyl- (BDM)	X	X	X	X	**91**, 106
**14.39**	Dipropylene glycol monomethyl ether (DPM)	–	–	–	X	**59**, 103
**18.22**	Dimethyl glutarate (DMG)	–	X	X	X	**59**, 100, 129
**21.00**	EA-MMA sesquimer (EA-MMA S)	X	–	–	X	**55**, 101, 128, 157
**21.84**	Diethyl glutarate (DEG)	X	–	–	–	55, 87, 115, **143**
**23.00**	1,4-Benzenedicarbonitrile (BDC)	X	X	–	X	101, **128**
**23.33**	EA-MMA dimer (EA-MMA D)	X	–	–	X	67, 95, **140**, 169
**24.99**	2,4,7,9-tetramethyl-5-decyne-4,7-diol (Surfynol 104) (S104)	–	–	–	X	**109**, 151, 169
**25.35**	3-Chloro-1,2-benzenedicarbonitrile (CBDC)	X	–	–	–	100, 127, **162**
**26.5-28.5**	1,6-Anhydro-β-D-glucopyranose (βDGP)	X	X	X	X	**60**, 73, 98
**28.95**	Octyl phenol (OP) - traces	X	–	–	–	**135**, 206
**29.07**	2,4-Dichloro-1,3-benzenedicarbonitrile (DBDC)	X	–	–	–	125, 161, **196**
**30.07**	MA trimer (MA T)	X	X	X	X	59, 79, **134**, 167, 194, 227

RT (min)	Compound	Green S510 (Schmincke AEROCOLOR)	Green RACBG (Rotring ArtiColor)	Green RACPG (Rotring ACP)	Blue WNPC (Winsor & Newton)	Main *m/z*
**30.8**	1,3,7-Trichloronaphthalene (TCN)	X	–	–	–	160, **230**
**33.24**	EA trimer (EA T)	X	–	–	X	79, 134, **208**, 255
**33.62**	Tetrachloroisophthalonitrile (TCIP)	X	–	–	–	109, 200, **266**
**35.30**	Palmitic acid (PA)	X	X	–	–	**73**, 129, 157, 213, 227, 256
**36.86**	Oleyl alcohol, methyl ether (OAME)	–	–	X	–	55, **82**, 96, 110, 124
**40.05**	Triethyleneglycol monododecyl ether (TEGMO)	–	–	X	–	**57**, 89, 124, 166
**43.90**	Bis(2-ethylhexyl) phthalate (DEHP) - traces	X	X	X	X	**149**, 167, 279

References

[1] V. Pintus, M. Schreiner, Characterization and identification of acrylic binding media: influence of UV light on the ageing process, Anal Bioanal Chem 399 (2011) 2961–2976. https://doi.org/10.1007/s00216-010-4357-5.

[2] T. Learner, The Analysis of Synthetic Paints by Pyrolysis-Gas Chromatography-Mass Spectrometry (PyGCMS), Studies in Conservation 46 (2001) 225. https://doi.org/10.2307/1506773.

[3] T. Learner, Analysis of Modern Paints, Getty Publications, 2004.

[4] Ș. Moldoveanu, Analytical pyrolysis of synthetic organic polymers, Elsevier, Amsterdam, 2005.

[5] E.A. Willneff, S.L. Schroeder, B.A. Ormsby, Spectroscopic techniques and the conservation of artists' acrylic emulsion paints, Herit Sci 2 (2014) 25. https://doi.org/10.1186/s40494-014-0025-y.

[6] C. Vanini, Solvent based inkjet ink formulation, WO2005116149A1, 2005. https://patents.google.com/patent/WO2005116149A1/en (accessed October 19, 2025).

[7] Ink jet ink composition, 2000. https://eureka.patsnap.com/patent-US6139611A (accessed October 19, 2025).

[8] SURFYNOL® 61, (n.d.). https://products.evonik.com/assets/or/ld/SURFYNOL_61_TDS_EN_EN_TDS_PV_52042436_en_GB_WORLD.pdf (accessed October 16, 2025).

[9] SURFYNOL® 104 DPM, (n.d.). https://products.evonik.com/assets/or/ld/SURFYNOL_104_DPM_TDS_EN_EN_TDS_PV_52042442_en_GB_WORLD.pdf (accessed October 16, 2025).

[10] Additives for Coatings, Inks, and Adhesives, (n.d.). https://www.chem-on.com.sg/image/catalog/Brochures/Brochure%20-%20SURFYNOL.pdf (accessed October 16, 2025).

[11] F. Liguori, C. Oldani, L. Capozzoli, N. Calisi, P. Barbaro, Liquid-phase synthesis of methyl isobutyl ketone over bifunctional heterogeneous catalysts comprising cross-linked perfluorinated sulfonic acid Aquivion polymers and supported Pd nanoparticles, Applied Catalysis A: General 610 (2021) 117957. https://doi.org/10.1016/j.apcata.2020.117957.

[12] Elvacite® 2046, (n.d.).

[13] E. Ghelardi, I. Degano, M.P. Colombini, J. Mazurek, M. Schilling, T. Learner, Py-GC/MS applied to the analysis of synthetic organic pigments: characterization and identification in paint samples, Anal Bioanal Chem 407 (2015) 1415–1431. https://doi.org/10.1007/s00216-014-8370-y.

[14] H. Van Keulen, M. Schilling, AMDIS & EXCEL: A Powerful Combination for Evaluating THM-Py-GC/MS Results from European Lacquers, Studies in Conservation 64 (2019) S74–S80. https://doi.org/10.1080/00393630.2019.1594580.
